# Impact of health warning labels on selection and consumption of food and alcohol products: systematic review with meta-analysis

**DOI:** 10.1080/17437199.2020.1780147

**Published:** 2020-07-02

**Authors:** Natasha Clarke, Emily Pechey, Daina Kosīte, Laura M. König, Eleni Mantzari, Anna K.M. Blackwell, Theresa M. Marteau, Gareth J. Hollands

**Affiliations:** a Behaviour and Health Research Unit, Institute of Public Health, University of Cambridge, Cambridge, UK; b Psychological Assessment & Health Psychology, Department of Psychology, University of Konstanz, Konstanz, Germany; c Tobacco and Alcohol Research Group, School of Psychological Science, University of Bristol, Bristol, UK

**Keywords:** Health warning labels, sugar sweetened beverages, alcohol, food, systematic review, meta-analysis

## Abstract

Health warning labels (HWLs) could reduce harmful consumption of food (including non-alcoholic drinks) and alcoholic drinks. A systematic review with meta-analysis using Cochrane methods was conducted to assess the impact on selection (including hypothetical selection) or consumption of food or alcoholic drink products displaying image-and-text (sometimes termed ‘pictorial’) and text-only HWLs. Fourteen randomised controlled trials were included, three for alcohol, eleven for food. For the primary outcomes, eleven studies measured selection and one measured consumption (two measured only other secondary outcomes). Meta-analysis of twelve comparisons from nine studies (n=12,635) found HWLs reduced selection of the targeted product compared with no HWL (RR=0.74 (95%CI 0.68–0.80)), with participants 26% less likely to choose a product displaying a HWL. A planned subgroup analysis suggested a larger (although not statistically significant) effect on selection of image-and-text HWLs (RR=0.65 (95%CI 0.54–0.80)) than text-only HWLs (RR=0.79 (95%CI 0.74–0.85)). These findings suggest significant potential for HWLs to reduce selection of food and alcoholic drinks, but all experimental studies to date were conducted in laboratory or online settings with outcomes assessed immediately after a single exposure. Studies in field and naturalistic laboratory settings are needed to estimate the potential effects of food and alcohol HWLs.

*Study registration***:** PROSPERO 2018 (registration number: CRD42018106522).

## Introduction

The harmful consumption of food, alcoholic and non-alcoholic drinks (principally sugar-sweetened beverages (SSBs)) are key preventable causes of non-communicable diseases including many cancers, heart disease and type 2 diabetes (Rehm et al., 2018; Sheron & Gilmore, 2016; Te Morenga et al., 2013). Reducing the significant public health burden of harmful consumption and promoting healthy lifestyles are key objectives of global and national public health bodies.

Altering cues in the proximal (small-scale) environments where individuals select and purchase products, often described as ‘choice architecture’ or ‘nudging’ interventions, has the potential to change harmful health-related behaviours (Cadario & Chandon, 2018; Hollands et al., 2013). One potential intervention to influence and reduce the harmful consumption of these products at the point of decision involves adding labels to product packaging, classed as an ‘Information’ intervention in the TIPPME typology for changing environments to change behaviour (Hollands et al., 2017). These types of information-based choice architecture interventions can shape behaviour, with recent systematic reviews showing their influence on selection and consumption of food and alcohol (Carter et al., 2018). Nutritional labelling, particularly calorie labels displayed on food (Crockett et al., 2018; Shangguan et al., 2019) and menus may also reduce energy purchased (Bleich et al., 2017). Health warning labels (HWLs) are another type of label that could be applied across populations. While their impact has been mainly studied in the context of tobacco (Brewer et al., 2016; Hammond, 2011), there is growing interest in their use for unhealthier foods and alcohol (Hollands et al., 2011; Mantzari et al., 2018; Wigg & Stafford, 2016). The current review focuses on the impact on selection or consumption of HWLs as applied to food (including non-alcoholic drinks) and alcohol.

HWLs communicate information that describes the potential adverse health consequences of excessive consumption of the product – such as an increased risk of disease – using an image with accompanying text (image-and-text HWL), or text alone (text-only HWL). Currently, 118 countries have adopted image-and-text (also known as ‘pictorial’ or ‘graphic’) HWLs on tobacco packaging, covering 58% of the world’s population (CCS, 2018). Evidence of their impact on smoking behaviours suggests that warnings displayed as text alone or with images reduce smoking (Hammond, 2011; Hammond et al., 2004). Compared to text-only HWLs, image-and-text HWLs are more effective (Hammond, 2011; Noar et al., 2016b). Strengthened warnings – defined as improvements to text-only warnings, the implementation of images alongside text or improved image warnings – increase perceived effectiveness outcomes (Noar et al., 2017), increase knowledge (i.e., of health effects) and show associations with increased quit attempts and decreased cigarette consumption (Noar et al., 2016a). The specific mechanisms through which HWLs change behaviour are unclear and warrant further research. A recent analysis of potential mechanisms underlying tobacco HWLs identified a number of mediators, including the elicitation of self-reported negative affect (i.e., fear, disgust), thinking about the warning and harms of smoking, and increased attention (Brewer et al., 2019). HWLs that generate negative emotions – such as fear, disgust and worry – increase the likelihood of quit attempts (Cho et al., 2018), lead to higher risk perceptions and stronger intentions to quit (Kees et al., 2010). These effects have been observed across socioeconomic groups suggesting HWLs are promising population-level interventions that would not increase health inequalities (Cantrell et al., 2013).

Given this evidence, the use of HWLs on other health-damaging products, specifically alcohol and foods high in fat, sugar and salt, has been proposed (Pomeranz et al., 2018; Smith & Al-Hamdani, 2017). A number of US states – including California, Baltimore and New York – have proposed the use of HWLs on sugary drinks (AHA, 2016). Many countries are enhancing front of pack food labelling, such as Chile (Reyes et al., 2019). There are also calls from public health bodies for improved labelling, including health warnings, on alcohol packaging (RSPH, 2017). It is unclear, however, whether their effectiveness extends beyond tobacco. Evidence of the impact of HWLs on the selection or consumption of food and alcohol is more limited. Initial evidence suggests that placing these labels on such products is relatively acceptable to the public (Gollust et al., 2014; Mantzari et al., 2018; Reynolds et al., 2019), which increases the likelihood of their implementation (Cullerton et al., 2016). Assessment of underlying mechanisms for food and alcohol HWLs is also largely absent. One of the few laboratory studies found that presenting aversive images of potential health consequences with snack foods impacted implicit and explicit attitudes towards those products and mediated reduced preferences for unhealthy snacks in choice tasks (Hollands & Marteau, 2016). While several studies have evaluated the impact of HWLs – including graphic and aversive image labels – on selection and consumption of food and alcohol (Billich et al., 2018; Mantzari et al., 2018; Stafford & Salmon, 2017), this evidence has yet to be quantitatively synthesised and appraised to enable a robust estimate of likely effects on these outcomes. There is one narrative synthesis of studies of HWLs on alcohol, but this mainly focused on non-behavioural outcomes, including attitudes and believability (Hassan & Shiu, 2018).

The primary aim of the current review is to estimate the impact of image-and-text or text-only HWLs, placed on product packaging, on selection (including purchasing) and consumption of food (including non-alcoholic drinks) and alcohol. A secondary aim is to estimate the impact of these HWLs on cognitive and emotional outcomes.

## Methods

A protocol was developed, following the PRISMA guidelines (Moher et al., 2009) and Cochrane methods described in the Cochrane Handbook for Systematic Reviews (Higgins et al., 2019). This was registered on the PROSPERO international Prospective Register of Systematic Reviews database in advance of the review being conducted (registration number CRD42018106522).

### Criteria for inclusion in the review

#### Types of studies

Studies were required to be randomised controlled trials or quasi-randomised controlled trials *i.e.,* controlled trials with a non-random method of allocation to study group such as alternation or by date of birth, with either between-subjects (parallel group) or within-subjects (cross-over) designs. Studies were required to compare at least two groups, one group comprising participants exposed to a HWL placed on food (including non-alcoholic drinks) or alcoholic drinks, and one group exposed to no label or any other non-health-related information, such as a barcode label. Studies were classified into one of three groups: *i.* online (web-based), *ii.* laboratory (attending in person in an artificial or naturalistic laboratory) or *iii.* field studies (attending in person in a ‘real-life’ setting). The distinction between online and laboratory settings was based on existing definitions of online studies (Finley & Penningroth, 2015).

#### Types of participants

Adults or children consuming products or selecting products for themselves, or selecting products on behalf of someone else, such as adults selecting for children.

#### Type of health warning label

HWLs were defined as labels containing an image accompanied by text (image-and-text HWL), or text alone (text-only HWL), describing one or more adverse health consequences to an individual or to others of consuming food (including non-alcoholic drinks) and alcohol. If the label displayed an image, it was required to contain a photographic or pictorial representation of the human body’s structure, anatomy or pathology and be accompanied by text describing the represented health consequence(s). Only studies that included labels that were placed on the product packaging were included.

#### Types of outcomes

##### Primary outcomes

Eligible outcomes were those assessing selection or purchasing of a product for consumption (including hypothetical selection), or consumption. Hypothetical selection outcomes were required to have a clear endpoint measured at the time of the behaviour being enacted, such as hypothetically selecting a product for immediate consumption. If there were multiple selection outcomes, selection at the level of the product was used, rather than a volume-based measure of nutrients or energy selected.

##### Secondary outcomes

Eligible outcomes were those assessing intention or motivation to change selection or consumption of the target product, or negative emotional responses (including fear, disgust, worry or discomfort). Where studies reported acceptability of HWLs, these data were extracted. This secondary outcome was the only outcome not specified in the protocol but was considered potentially informative to research and policy in this area.

### Exclusion criteria

We excluded studies where HWLs were not placed directly on the product of interest, e.g., information-based cues placed at point of purchase such as on posters or flyers, and those where HWLs warned only of the product’s contents such as energy (kcal) or alcohol content (% alcohol by volume).

#### Search strategy

An electronic search strategy was developed which included free-text terms based on the eligibility criteria *e.g.,* ‘warning’, ‘message’, ‘graphic’, ‘label’, ‘drink’, ‘eat’, ‘snack’, ‘alcohol’, and, where possible, controlled vocabulary (e.g., MeSH) terms. The search was initially developed for MEDLINE (OvidSP In-Process 1946 to 16th September 2019). The search strategy was then adapted for the following databases: Embase (OvidSP) (1980 to 16th September 2019), PsycINFO (EBSCO) (1806 to 16th September 2019), Cochrane Central Register of Controlled Trials (CENTRAL) (1992 to 16th September 2019), Science Citation Index Expanded (Web of Science) (1900 to 16th September 2019), Social Sciences Citation Index (Web of Science) (1956 to 16th September 2019), Conference Proceedings Citation Index – Science (Web of Science) (1990 to 16th September 2019), Conference Proceedings Citation Index – Social Science & Humanities (Web of Science) (1990 to 16th September 2019) and PsyArXiv (7th October 2019) (see Supplementary material 3 for all searches). The search was limited to reports in the English language. There was no restriction on publication date. On two separate occasions (most recent search: 9th October 2019) the reference lists of all eligible study reports were searched and forward citation tracking conducted (using Google Scholar) to identify further eligible studies or study reports. For the grey literature, in addition to the aforementioned Web of Science searches of two Conference Proceedings databases, we conducted a search in PsyArXiv (a repository of preprint articles from the psychological sciences), which closely mapped onto terms used in the main search strategy.

#### Study selection

All records retrieved by the electronic searches were exported to a reference manager (Endnote X8) to facilitate screening. Duplicates were removed and abstracts were screened against the eligibility criteria by two reviewer authors, working independently. Title and abstract records were coded as provisionally eligible or excluded. Any disagreements in the coding of the title and abstract records were identified and resolved by discussion to reach a consensus between the two review authors, with a third author acting as arbiter if consensus was not reached.

**Figure 1.**
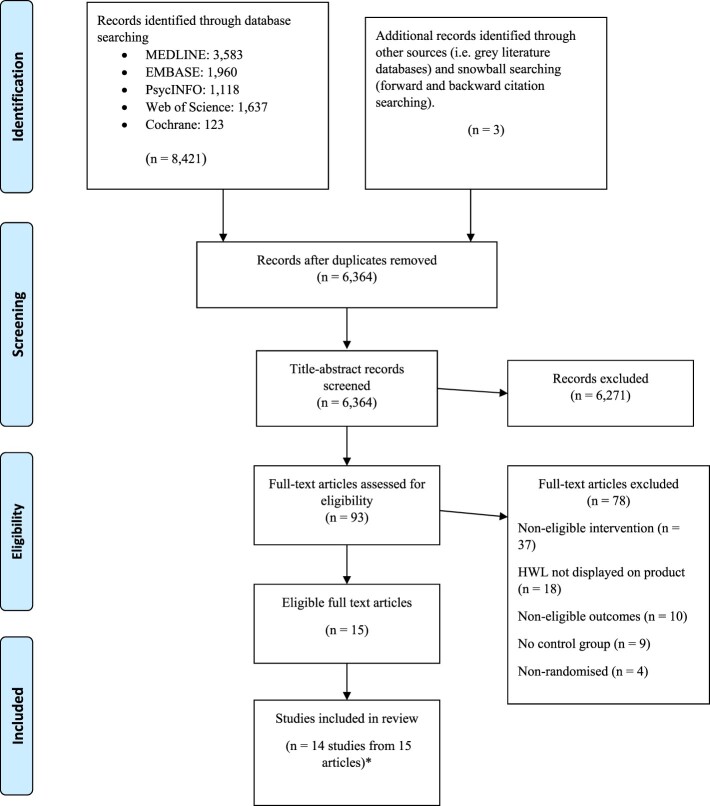
PRISMA flow diagram.

Full-text reports were obtained for all records coded as ‘provisionally eligible’. Screening of full-text study reports was undertaken by two review authors working independently. Full-text study reports were coded as ‘eligible’ or ‘excluded’, with the reasons for exclusion recorded. Any disagreements in the coding of the full-text records was identified and resolved by discussion to reach a consensus between the two review authors, with a third author acting as arbiter if consensus was not reached. A PRISMA flow diagram (Moher et al., 2009) documented the flow of records and studies (see Figure 1).

Study characteristics and outcome data were extracted by two review authors working independently. Any discrepancies in the extracted data were identified and resolved by discussion to reach a consensus between the two review authors. If they were unable to reach a consensus a third author acted as arbiter. Study authors were contacted to obtain key unpublished primary outcome data.

A data extraction form was developed and the following key data were extracted from each included study:

- Lead author
- Date
- Participant inclusion criteria
- Study population
- Study design
- Study setting
- Product category
- Label type and content
- Number of participants in each group
- Outcome measures
- Duration of exposure
- Results
- Additional comments

#### Risk of bias

Risk of bias in the included studies was assessed for the primary outcomes using the Cochrane ‘Risk of bias 2.0’ tool for randomised trials (RoB 2.0) (Sterne et al., 2019). RoB 2.0 addresses five specific domains: *i.* bias arising from the randomisation process; *ii.* bias due to deviations from intended interventions; *iii.* bias due to missing outcome data; *iv.* bias in measurement of the outcome; and, *v.* bias in selection of the reported result. The tool was applied to each included study by two review authors working independently. Supporting information and judgements for risk of bias was provided for each domain (*low, high, some concerns*). Where possible, this was supported by verbatim text extracted from study reports. Any discrepancies in judgements of risk of bias were identified and resolved by discussion between two review authors, with a third author acting as arbiter for unresolved discrepancies. An overall summary ‘Risk of bias’ judgement (*low, high, some concerns*) for each study was derived based on the included domains. The overall summary judgement for each study was determined by the highest risk of bias level in any of the domains that were assessed. For example, a study was only considered to have summary ‘low risk’ of bias if all domains were judged as ‘low risk’. If any one domain was judged as ‘some concerns’, the overall summary was judged as ‘some concerns’, and if any one domain was judged as ‘high risk’, the overall summary was judged as ‘high risk’ of bias.

The risk of bias assessment was considered when determining the strength of results of the data synthesis, in developing conclusions and any recommendations concerning the design and conduct of future research.

#### Synthesis

A narrative synthesis of the included studies was conducted, presenting their major characteristics and results. Studies were judged to be sufficiently similar in their characteristics given our pre-specified eligibility criteria to enable data to be pooled statistically from studies for which there was more than one comparison for the outcome. Meta-analyses were conducted in Review Manager 5.3. Random-effects meta-analysis was used to obtain a pooled effect size with 95% CIs, with a Relative Risk (RR) as the effect size for dichotomous data (Mantel- Haenszel method) and a Standardised Mean Difference (SMD) for continuous data (Inverse Variance method). Random-effects models were used due to expected heterogeneity in study characteristics such as settings and participants. For studies of multi-component interventions that used factorial designs, providing each of the respective groups met our inclusion criteria and there were no interactions between factors, we combined outcome data across groups to capture the effect attributable to the HWL comparison. Where there was evidence of an interaction between factors, studies were analysed using only outcome data from those groups that represented the purest specific HWL comparison of interest to preclude possible confounding.

For the single study using a within-subjects design (Temple et al., 2016) study results were reported narratively, as insufficient detail was provided by study authors to enable inclusion in the meta-analysis, in line with Cochrane guidance (Becker & Balagtas, 1994; Higgins et al., 2019).

#### Certainty of evidence

GRADE (Grading of Recommendations, Assessment, Development and Evaluations) framework (Guyatt et al., 2011) was used to rate the certainty of each body of evidence relating to primary outcomes that were incorporated into meta-analyses, to indicate the confidence that can be placed in summary estimates of effect. This is an assessment of the likelihood that the true effect will not differ substantially from the estimated effect. Within the GRADE approach, the certainty of a body of evidence for intervention effects is assessed based on the design of the underlying studies and on a number of factors that can decrease or increase certainty. GRADE criteria for downgrading certainty of evidence encompass study limitations, inconsistency, imprecision, indirectness, publication bias and other considerations.

### Results

#### Results of the search

The search strategy detected 6,364 unique records. Abstract and title screening identified 93 full-text articles as potentially eligible for inclusion. Fifteen articles (which included 14 studies as two articles reported the same study) met all inclusion criteria and were included in the review. Details are shown in the PRISMA flow diagram (Figure 1) (Moher et al., 2009).

#### Characteristics of included studies

Fourteen studies were included in the review, providing data from 13,725 participants (Acton & Hammond, 2018; Ang et al., 2019; Billich et al., 2018; Bollard et al., 2016; Clarke et al., 2020a, 2020b; Grummon et al., 2019; Mantzari et al., 2018; Mantzari et al., 2020; Roberto et al., 2016; Stafford & Salmon, 2017; Temple et al., 2016; VanEpps & Roberto, 2016; Wigg & Stafford, 2016). Six of the studies were conducted in the UK (Clarke et al., 2020a, 2020b; Mantzari et al., 2018; Mantzari et al., 2020; Stafford & Salmon, 2017; Wigg & Stafford, 2016), four in the US (Grummon et al., 2019; Roberto et al., 2016; Temple et al., 2016; VanEpps & Roberto, 2016), one in Australia (Billich et al., 2018), one in Canada (Acton & Hammond, 2018), one in Singapore (Ang et al., 2019) and one in New Zealand (Bollard et al., 2016). Six of the studies targeted adults in the general population (Ang et al., 2019; Billich et al., 2018; Clarke et al., 2020a, 2020b; Grummon et al., 2019; Mantzari et al., 2020), one targeted university students (Wigg & Stafford, 2016), one targeted female university students (Stafford & Salmon, 2017), three targeted adolescents and young adults (Acton & Hammond, 2018; Bollard et al., 2016; Temple et al., 2016), one targeted adolescents only (VanEpps & Roberto, 2016), and two targeted parents (Mantzari et al., 2018; Roberto et al., 2016). Two studies over-sampled Hispanics and African Americans (Roberto et al., 2016; VanEpps & Roberto, 2016), stating the reason as this group having the highest obesity prevalence in the US. All studies were conducted in artificial settings: eight were conducted online, with participants completing the study via a web-based platform (Ang et al., 2019; Billich et al., 2018; Bollard et al., 2016; Clarke et al., 2020a, 2020b; Mantzari et al., 2018; Roberto et al., 2016; VanEpps & Roberto, 2016), and six were conducted in laboratory settings, with participants attending study sessions in person (Acton & Hammond, 2018; Grummon et al., 2019; Mantzari et al., 2020; Stafford & Salmon, 2017; Temple et al., 2016; Wigg & Stafford, 2016). One of the laboratory studies was conducted in a naturalistic convenience store laboratory (Grummon et al., 2019). See Table 1 for full study characteristics.

**Table 1.** Characteristics of Included Studies.

Author reference and country	Design	Study setting	Population		Product category	Label type (i.e., text-only, image-and-text)	Content of label	Comparisons included in the review	Outcome measures	Duration	Results (primary outcomes)
Acton and Hammond (2018) Canada	Between-subjects RCT	Laboratory		Young adults 16 +	Non-alcoholic drinks (SSBs)	Text-only	‘WARNING: Drinking beverages with added sugar (s) contributes to obesity, diabetes and tooth decay.’	No label vs. text-only HWL	Primary: Selection (selection of SSB with purchase)	Immediate (≤ one day)	No significant effect of text-only HWL on outcomes compared to control condition
Ang et al. (2019) Singapore	Between-subjects RCT	Online	Adults 21+		Food and non-alcoholic drinks (SSBs and high-in-sugar food)	Text-only	‘HEALTH WARNING: Consuming products with added sugar(s) contributes to obesity, diabetes, and tooth decay.’	No label vs. text-only HWL	Primary: Selection (proportion of high in sugar products selected, with purchase)	Immediate (≤ one day)	The proportion of high in sugar products selected was lower in the text-only HWL group compared to the control arm
Billich et al. (2018) Australia	Between-subjects RCT	Online	Adults 18–35		Non-alcoholic drinks (SSBs)	Text-only and image-and-text	Text-only HWL: ‘Warning: Drinking drinks with added sugar contributes to obesity, type 2 diabetes and tooth decay’. Image-and-text HWL: The addition of an image showing rotting teeth.	No label vs. text-only HWL vs. image-and-text HWL	Primary: Selection (proportion of participants selecting sugary drink)	Immediate (≤ one day)	Compared to the control group, the image-and-text HWL, text-only HWL, sugar information and HSR labels all significantly reduced selection of a SSB in the choice scenario. The magnitude of effect was greatest for the image-and-text HWL
Bollard et al. (2016) New Zealand	Between-subjects RCT	Online	Adolescents and young adults 13–24		Non-alcoholic drinks (SSBs)	Text-only and image-and-text	Text-only HWL: ‘WARNING: Drinking beverages with added sugar(s) contributes to obesity, diabetes, and tooth decay’ Image & text HWL: The addition of an image showing tooth decay.	No label vs. text-only HWL vs. image-and-text HWL	Secondary: Intention to purchase SSB		-
Clarke et al. (2020a) UK	Between-subjects RCT	Online	Adults 18+		Alcoholic drinks (beer and wine)	Text-only and image-and-text	Text-only HWL: ‘Excess calories cause [liver cancer, bowel cancer, breast cancer]’ Image-and-text HWL: the addition of an image showing bowel cancer, liver cancer, breast cancer (diseased organs or surgery scar)	No label vs. text-only HWL vs. image-and-text HWL	Primary: Selection (proportion of participants selecting alcoholic beverage) Secondary: Negative emotional arousal, acceptability	Immediate (≤ one day)	Text-only and image-and-text HWLs significantly reduced selection of alcoholic drinks compared to no label
Clarke et al. (2020b) UK	Between-subjects RCT	Online	Adults 18+		Food (energy-dense snacks)	Text-only and image-and-text	Text-only HWL: ‘Excess calories cause obesity, which causes [heart disease, bowel cancer, type 2 diabetes]’ Image-and-text HWL: the addition of an image showing bowel cancer, heart disease, type 2 diabetes (diseased organs and blinded eye)	No label vs. text-only HWL vs. image-and-text HWL	Primary: Selection (proportion of participants selecting energy-dense snack) Secondary: Negative emotional arousal, acceptability	Immediate (≤ one day)	Text-only and image-and-text HWLs significantly reduced selection of energy-dense snacks compared to no label
Grummon et al. (2019) USA	Between-subjects RCT	Laboratory	Adults 18+		Non-alcoholic drinks (SSBs)	Text-only	‘WARNING. Beverages with added sugar contribute to tooth decay, diabetes, and obesity’	Barcode label vs. text-only HWL	Primary: Selection (selection of SSB with purchase) Secondary: Negative emotional arousal, intentions to limit consumption, acceptability	Immediate (≤ one day)	Text-only HWLs reduced SSB purchases
Mantzari et al. (2018) UK	Between-subjects RCT	Online	Parents selecting for children age 11–16		Non-alcoholic drinks (SSBs)	Image-and-text	An image of rotting teeth alongside the caption ‘Excess sugar intake causes dental decay’	No label vs. image-and-text HWL	Primary: Selection (selection of SSB) Secondary: Negative emotional arousal, acceptability	Immediate (≤ one day)	Image-and-text HWLs significantly reduced selection of SSBs compared to control labels
Mantzari et al. (2020) UK	Between-subjects RCT	Laboratory	Adults		Non-alcoholic drinks (SSBs)	Image-and-text	An image of rotting teeth alongside the caption ‘Excess sugar consumption causes dental decay’	No label vs. image-and-text HWL	Primary: Selection (selection of SSB)	Immediate (≤ one day)	Addition of an image-and-text HWL or calorie information label on SSB packaging did not reduce selection of SSBs
Roberto et al. (2016) USA	Between-subjects RCT	Online	Primary caregiver of child age 6–11		Non-alcoholic drinks (SSBs)	Text-only	4 HWL conditions: California label: 'drinking beverages with added sugar (s) contributes to obesity, diabetes and tooth decay'; Weight gain label: 'drinking beverages with added sugar (s) contributes to weight gain, diabetes and tooth decay' Preventable label: 'drinking beverages with added sugar (s) contributes to preventable diseases like obesity, diabetes and tooth decay' Type 2 diabetes label: 'drinking beverages with added sugar (s) contributes to obesity, type 2 diabetes and tooth decay'	No label vs. 4 text-only HWLs combined	Primary: Selection (selection of SSB) Secondary: Acceptability, intentions to purchase	Immediate (≤ one day)	Caregivers who saw SSBs with text-only HWLs were significantly less likely to choose an SSB relative to those who saw calorie or no labels on beverages
Stafford and Salmon (2017) UK	Between-subjects RCT	Laboratory	Adults (students)		Alcoholic drinks (alcopops)	Text-only and image-and-text	Text-only HWL: 'alcohol causes fatal liver cancer' Image-and-text HWL: The addition of an image showing a diseased liver.	No label vs. text-only HWL vs. image-and-text HWL	Primary: Consumption (consumption speed) Secondary: Acceptability	Immediate (≤ one day)	Alcohol was consumed at a faster rate for those in the control condition compared to both the image-and-text HWL and text-only conditions
Temple (2016) USA	Within-subjects RCT	Laboratory	Young adults 15–30		Non-alcoholic drinks (caffeinated energy drinks)	Text-only	Caffeine HWL: 'high levels of caffeine intake can cause headache, nausea, anxiety, irregular heartbeat, vomiting, and, in extreme cases, death. Use caution when consuming caffeine'	No label vs. text-only HWL	Primary: Selection (selection of ED with purchase)	Immediate (≤ one day)	The adolescent population may be sensitive to labelling, but labelling would not have an impact among adult ED consumers
VanEpps and Roberto (2016) USA	Between-subjects RCT	Online	Adolescents 12–18		Non-alcoholic drinks (SSBs)	Text-only	4 HWL conditions: California label: 'drinking beverages with added sugar (s) contributes to obesity, diabetes and tooth decay' Weight gain label: 'drinking beverages with added sugar (s) contributes to weight gain, diabetes and tooth decay' Preventable label: 'drinking beverages with added sugar (s) contributes to preventable diseases like obesity, diabetes and tooth decay' Type 2 diabetes label: 'drinking beverages with added sugar (s) contributes to obesity, type 2 diabetes and tooth decay'	No label vs. 4 text-only HWLs combined	Primary: Selection (selection of SSB) Secondary: Intentions to purchase, acceptability	Immediate (≤ one day)	Participants who saw SSBs with text-only HWLs were less likely to hypothetically purchase an SSB relative to those who saw no labels, an effect that was statistically significant for three of four label conditions
Wigg and Stafford (2016) UK	Between-subjects RCT	Laboratory	Adults (students)		Alcoholic drinks (beer and wine)	Text-only and image-and-text	Text-only HWL: 'alcohol causes fatal liver cancer' Image-and-text HWL: The addition of an image showing a diseased liver.	No label vs. text-only HWL vs. image-and-text HWL	Secondary: Intention to quit; negative emotional arousal (fear)	Immediate (≤ one day)	-

SSBs = sugar-sweetened beverages; HWL = health warning label; RCT = randomised controlled trial.

#### Types of studies and interventions

All studies were individually randomised controlled trials. Thirteen studies used a between-subjects design (Acton & Hammond, 2018; Ang et al., 2019; Billich et al., 2018; Bollard et al., 2016; Clarke et al., 2020a, 2020b; Grummon et al., 2019; Mantzari et al., 2018; Mantzari et al., 2020; Roberto et al., 2016; Stafford & Salmon, 2017; VanEpps & Roberto, 2016; Wigg & Stafford, 2016) and one used a within-subjects design (Temple et al., 2016). The majority of studies also compared other label groups (e.g., calorie labels, sugar content labels) in addition to HWLs. Thirteen studies had a ‘no label’ control condition and one study had a barcode image for the control condition (Grummon et al., 2019). Findings from two studies were collapsed into two-group comparisons (*i.e.,* HWL *vs* control). In these two studies, four variations of a text-only HWL were collapsed into a two-group comparison of text-only HWL *vs* control (Roberto et al., 2016; VanEpps & Roberto, 2016). Two studies investigated calorie labels in combination with HWLs (Clarke et al., 2020b; Mantzari et al., 2018). In one study, a significant interaction was reported between HWL and calorie information (Clarke et al., 2020b) and in the other study there was evidence of a potential interaction between HWL and calorie conditions (Mantzari et al., 2018). For both of these studies, groups in which additional calorie information was added to the HWL were excluded and a two-group comparison (HWL vs no HWL) was used in the analysis.

Five studies (Ang et al., 2019; Billich et al., 2018; Clarke et al., 2020b; Roberto et al., 2016; VanEpps & Roberto, 2016) also investigated other health-related information label conditions – calorie only, health star, sugar warning, image-only HWL – which were not eligible for inclusion in the analysis. Three studies (Acton & Hammond, 2018; Bollard et al., 2016; Temple et al., 2016) also investigated tax changes, pricing or plain packaging, in combination with HWL conditions. In two studies (Acton & Hammond, 2018; Temple et al., 2016) these additional interventions were consistently applied across HWL conditions, *i.e.,* the additional interventions were included as within-subject factors and participants in each HWL group completed each tax and price condition. In one study (Bollard et al., 2016) these additional interventions were between-subject factors (tax and plain packaging).

#### Target products

Nine of the fourteen studies targeted non-alcoholic drinks only (Acton & Hammond, 2018; Billich et al., 2018; Bollard et al., 2016; Grummon et al., 2019; Mantzari et al., 2018; Mantzari et al., 2020; Roberto et al., 2016; Temple et al., 2016; VanEpps & Roberto, 2016). Of these, eight targeted sugar-sweetened beverages (Acton & Hammond, 2018; Billich et al., 2018; Bollard et al., 2016; Grummon et al., 2019; Mantzari et al., 2018; Mantzari et al., 2020; Roberto et al., 2016; VanEpps & Roberto, 2016) and one targeted caffeinated energy drinks (Temple et al., 2016). One study targeted food products, specifically energy-dense snacks (Clarke et al., 2020b). One study targeted both food and non-alcoholic drinks (Ang et al., 2019). Three studies targeted alcohol products (Clarke et al., 2020a; Stafford & Salmon, 2017; Wigg & Stafford, 2016).

#### Characteristics of the HWLs

Twelve of the studies investigated text-only labels (Acton & Hammond, 2018; Ang et al., 2019; Billich et al., 2018; Bollard et al., 2016; Clarke et al., 2020a, 2020b; Grummon et al., 2019; Roberto et al., 2016; Stafford & Salmon, 2017; Temple et al., 2016; VanEpps & Roberto, 2016; Wigg & Stafford, 2016), with six of these also investigating image-and-text HWLs (Billich et al., 2018; Bollard et al., 2016; Clarke et al., 2020a, 2020b; Stafford & Salmon, 2017; Wigg & Stafford, 2016). Two studies investigated image-and-text HWLs only (Mantzari et al., 2018; Mantzari et al., 2020).

For sugar-sweetened beverages (Acton & Hammond, 2018; Ang et al., 2019; Billich et al., 2018; Bollard et al., 2016; Grummon et al., 2019; Mantzari et al., 2018; Mantzari et al., 2020; Roberto et al., 2016; VanEpps & Roberto, 2016) and sugary foods (Ang et al., 2019) the adverse health consequences described in the text of the HWLs focussed on health risks associated with sugar consumption, specifically obesity, diabetes (sometimes specified as Type 2) and tooth decay. For image-and-text HWLs, the image depicted tooth decay (Billich et al., 2018; Bollard et al., 2016; Mantzari et al., 2018; Mantzari et al., 2020). The text-only HWL on caffeinated drinks described short terms effects including headache, nausea, vomiting and long term effects, including anxiety, irregular heartbeat and in extreme cases, death (Temple et al., 2016). Alcohol text-only and image-and-text HWLs described liver cancer (Clarke et al., 2020a; Stafford & Salmon, 2017; Wigg & Stafford, 2016), breast and bowel cancers (Clarke et al., 2020a). Energy-dense snack image-and-text and text-only HWLs focussed on heart disease, type 2 diabetes and bowel cancer (Clarke et al., 2020b). The text used in labels is given in Table 1. Images used in each study are included in supplementary material (S4).

In nine of the studies (Acton & Hammond, 2018; Bollard et al., 2016; Clarke et al., 2020a, 2020b; Grummon et al., 2019; Mantzari et al., 2020; Stafford & Salmon, 2017; Temple et al., 2016; Wigg & Stafford, 2016) the HWLs were displayed on the physical product or image of the product. In five of the studies (Ang et al., 2019; Billich et al., 2018; Mantzari et al., 2018; Roberto et al., 2016; VanEpps & Roberto, 2016) – all conducted in online settings – the HWLs were also displayed enlarged, above, next to or below the product image, to ensure the HWL was large enough to view on a computer screen.

#### Outcome measures

##### Primary outcomes

Ten of the included studies measured product selection in which participants selected one or more products *i.e.,* a selection task in which one or more products were chosen out of a range of products, including the target product and also alternative ‘healthier’ options (Acton & Hammond, 2018; Billich et al., 2018; Clarke et al., 2020a, 2020b; Grummon et al., 2019; Mantzari et al., 2018; Mantzari et al., 2020; Roberto et al., 2016; Temple et al., 2016; VanEpps & Roberto, 2016). One study measured selection as the proportion of unhealthy choices (Ang et al., 2019). Four of the eleven studies measuring selection provided participants with money to spend (*i.e.,* purchasing) (Acton & Hammond, 2018; Ang et al., 2019; Grummon et al., 2019; Temple et al., 2016). One study measured speed of consumption of alcohol (Stafford & Salmon, 2017), an outcome that might predict volume consumption. None of the included studies measured volume of food or drink consumed.

##### Secondary outcomes

Six of the included studies with eligible primary outcomes also reported assessing eligible secondary outcomes (Clarke et al., 2020a, 2020b; Grummon et al., 2019; Mantzari et al., 2018; Roberto et al., 2016; VanEpps & Roberto, 2016;) and two studies reported secondary outcomes only (Bollard et al., 2016; Wigg & Stafford, 2016). Four studies (Clarke et al., 2020a, 2020b; Grummon et al., 2019; Mantzari et al., 2018) measured negative emotional arousal. One study measured fear arousal, a component of negative emotional arousal (Wigg & Stafford, 2016). Purchase or consumption intentions were measured in five studies (Bollard et al., 2016; Grummon et al., 2019; Roberto et al., 2016; VanEpps & Roberto, 2016; Wigg & Stafford, 2016). Five studies measured acceptability of HWLs (Clarke et al., 2020a, 2020b; Mantzari et al., 2018; Roberto et al., 2016; VanEpps & Roberto, 2016).

### Risk of bias assessment

For the overall summary risk of bias assessment for studies reporting primary outcomes, the majority of studies were judged to be subject to significant risk of bias *i.e.,* categorised as *some concerns* for the summary risk of bias (Sterne et al., 2019). For the majority of studies there was insufficient information provided in the published articles in some domains to enable judgements for signalling questions other than ‘No information’. For example, many studies were judged to have risk of bias for selective reporting of results, mainly due to not reporting pre-registered study protocols and analysis plans. In addition, many of the lab-based studies did not provide sufficient information on participant or researcher blinding or randomisation procedures. See Table 2 for RoB summary of articles, with more detail on key elements provided in the Supplementary material (2). A link to the complete set of extracted data can be found on the PROSPERO website (registration number: CRD42018106522).

**Table 2.** Risk of Bias Summary for Included Studies.

Study	Bias arising from the randomisation process	Bias due to deviations from intended interventions	Bias due to missing outcome data	Bias in measurement of the outcome	Bias in selection of the reported result	Overall risk of bias (selection)	Overall risk of bias (consumption)
Ang et al. (2019)	Low risk	Low risk	Low risk	Low risk	Some concerns	Some concerns	
Acton and Hammond (2018)	Some concerns	Low risk	Low risk	Some concerns	Some concerns	Some concerns	
Billich et al. (2018)	Some concerns	Low risk	Low risk	Low risk	Some concerns	Some concerns	
Clarke et al. (2020a)	Low risk	Low risk	Low risk	Low risk	Low risk	Low risk	
Clarke et al. (2020b)	Low risk	Low risk	Low risk	Low risk	Low risk	Low risk	
Grummon et al. (2019)	Low risk	Low risk	Low risk	Low risk	Low risk	Low risk	
Mantzari et al. (2018)	Low risk	Low risk	Low risk	Low risk	Some concerns	Some concerns	
Mantzari et al. (2020)	Low risk	Low risk	Low risk	Some concerns	Some concerns	Some concerns	
Roberto et al. (2016)	Low risk	Low risk	Low risk	Low risk	Some concerns	Some concerns	
Stafford and Salmon (2017)	Some concerns	Low risk	Low risk	Some concerns	Some concerns		Some concerns
Temple et al. (2016)	Some concerns	Some concerns	Low risk	Low risk	Some concerns	Some concerns	
VanEpps and Roberto (2016)	Low risk	Low risk	Low risk	Low risk	Some concerns	Some concerns	

### Effects of interventions

#### Primary outcomes

##### Selection

**Figure 2.**
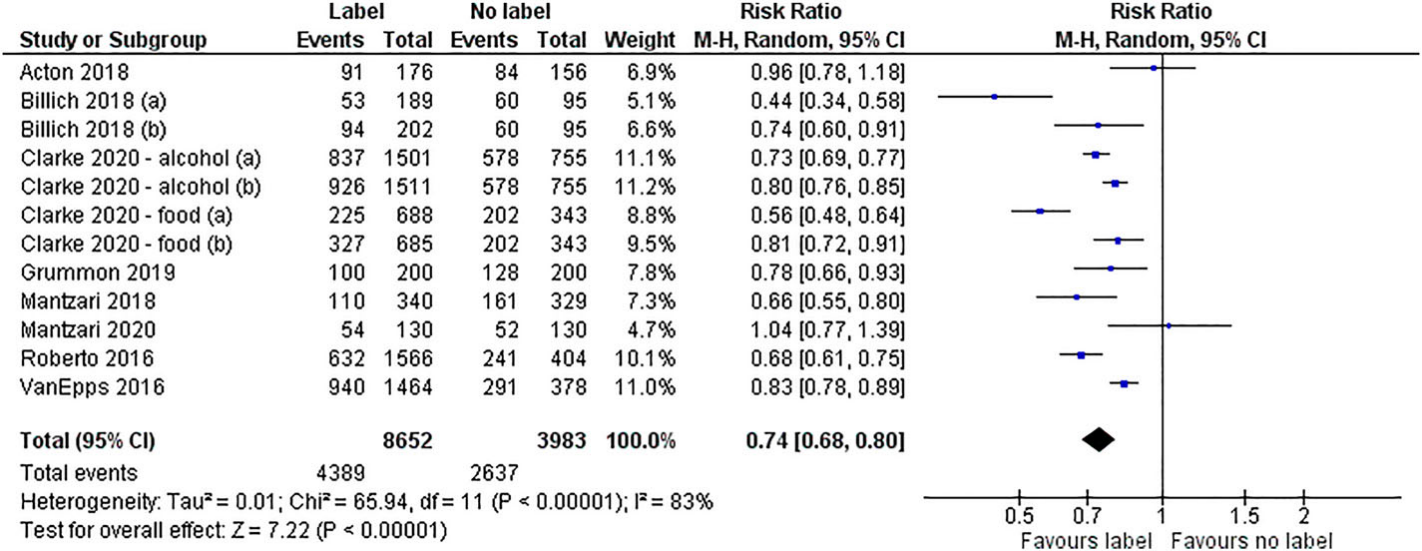
Forest plot of selection outcomes.

Dichotomous data were used from nine studies (Acton & Hammond, 2018; Billich et al., 2018; Clarke et al., 2020a, 2020b; Grummon et al., 2019; Mantzari et al., 2018; Mantzari et al., 2020; Roberto et al., 2016; VanEpps & Roberto, 2016). All of these studies measured product selection in which participants selected one product from a range of the targeted less healthy product and healthier alternatives. Pooled analysis of these data – comprising 12 comparisons from 9 studies (12,635 participants) – showed that HWLs had a large effect on reducing selection of the less healthy product: RR = 0.74 (95% CI, 0.68–0.80) (Figure 2). There was substantial statistical heterogeneity between studies (I^2 ^= 83%) with participants being 26% less likely to choose a product displaying a HWL. One study was excluded from the pooled analysis as it provided insufficient detail (Temple et al., 2016), not reporting differences in selection between warning labels groups. A further study that reported a selection outcome (Ang et al., 2019) used a continuous measure of selection and so was not included in the meta-analysis of dichotomous data. This study reported a reduction in the proportion of products that were high in sugar selected in the HWL group compared to the control group (*p* < 0.05).

##### GRADE assessment

Using the GRADE framework, the certainty of the evidence for the selection outcome was assessed as low meaning that including further studies is likely to change the estimate. The current evidence included in the meta-analysis was rated down by one level *i.e.,* judged as having serious limitations because the majority of study-level estimates of this effect were judged to have significant concerns for risk of bias. It was not rated down for imprecision, as the confidence intervals were extremely narrow and did not include the possibility of a meaningfully different effect, and the number of participants (sample size) incorporated into this meta-analysis was very large, exceeding the number generated by a conventional sample size calculation for a single adequately powered trial, powered conservatively to detect a small effect size. Although statistical heterogeneity was considerable, the evidence was not rated down for inconsistency because effect sizes were predominantly in a consistent direction, and the meta-analysis result was driven by large studies with considerable overlap in their typically precise effects. The evidence was rated down once for indirectness because only a small number of studies used HWLs applied to real products and there were no trials in field settings. Finally, the evidence was not rated down for other considerations including publication bias because there was no clear evidence of such bias in addition to there being insufficient studies to conduct formal assessment.

##### Subgroup analyses

**Figure 3.**
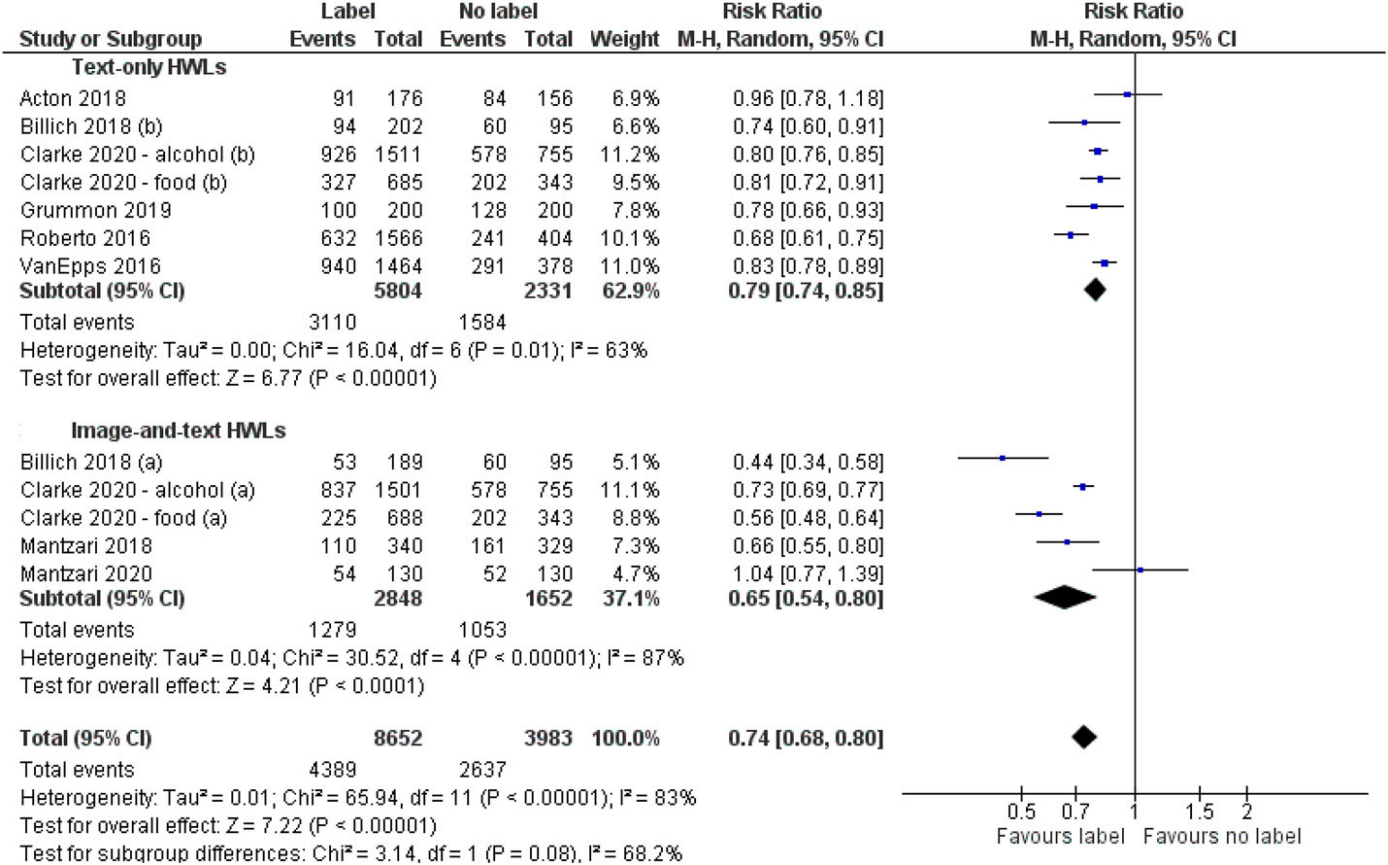
Forest plot of selection outcomes by HWL type.

**Figure 4.**
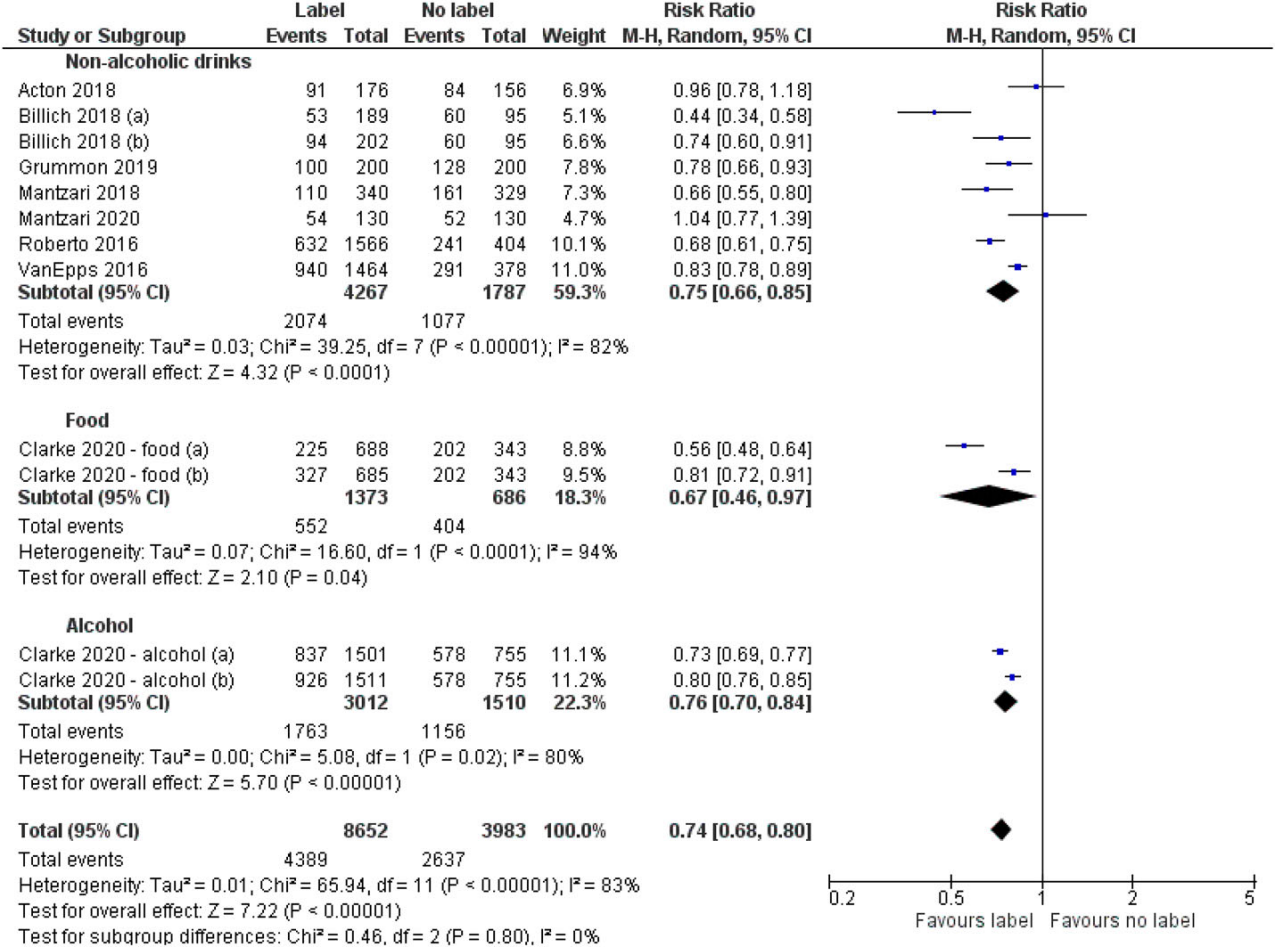
Forest plot of selection outcomes by product type.

Two pre-specified subgroup analyses were conducted concerning first, HWL type, and second, product type. There was a larger effect on selection of image-and-text HWLs (RR = 0.65 (95% CI, 0.54–0.80)) than text-only HWLs (RR = 0.79 (95% CI, 0.74–0.85)) (Figure 3) although this difference was not statistically significant (*p* = 0.08). Participants were 35% less likely to select a product with an image-and-text HWL compared to no label, and 21% less likely to select a product with a text-only HWL compared to no label. There were no differences in effects (*p* = 0.80) by product type: non-alcoholic drinks (RR = 0.75 (95% CI, 0.66–0.85)), food (RR = 0.67 (95% CI, 0.46–0.97)), alcoholic drinks (RR = 0.76 (95% CI, 0.70–0.84)) (Figure 4).

**Figure 5.**
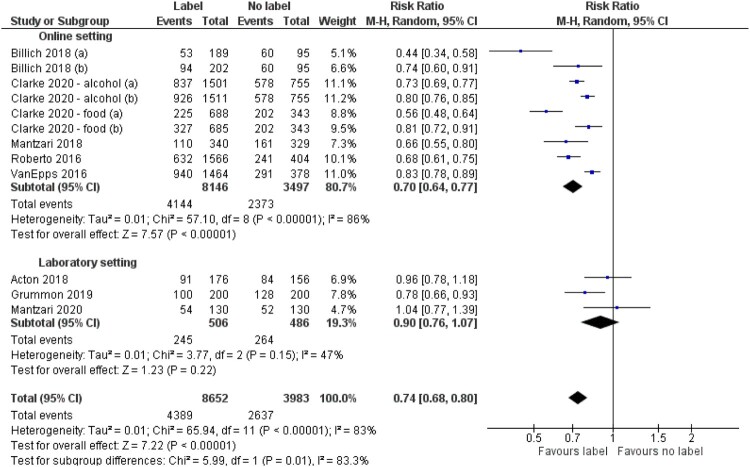
Forest plot of selection outcomes by setting.

A third, exploratory, subgroup analysis was conducted to interrogate the considerable observed heterogeneity (I^2^ = 83%) for the meta-analysis of the selection outcome. The visual pattern of results strongly suggested that study setting may underlie this heterogeneity: two of the three studies conducted in laboratory settings suggested no effect of HWLs while those conducted online reported substantial effects favouring the intervention, all with RR point estimates between 0.34–0.91. This was formally assessed in a subgroup analysis, finding differential effects (*p* = 0.01) between studies conducted in online settings using images of products (RR = 0.70 (95% CI, 0.64–0.77)), compared to laboratory settings using physical products (RR = 0.90 (95% CI, 0.76–1.07)) (Figure 5). Heterogeneity was considerably lower in the laboratory setting subgroup (I^2^ = 47%), heterogeneity remained high in the online setting subgroup (I^2^ = 86%), due to the inflated I^2^ resulting from precise estimates linked to large sample sizes (Rücker et al., 2008).

##### Consumption

Meta-analysis was not possible as there was only one study assessing an index of consumption (Stafford & Salmon, 2017). This investigated the impact of image-and-text and text-only labels on consumption speed, finding a statistically significant effect of HWL: consumption was slower in the text-only and image-and-text HWL conditions *vs.* control (*p* < .001). There were no significant differences between the two HWL conditions.

#### Secondary outcomes

##### Negative emotional arousal

Four studies (Clarke et al., 2020a, 2020b; Grummon et al., 2019; Mantzari et al., 2018) reported negative emotional arousal. One study measured fear arousal, a component of negative emotional arousal (Wigg & Stafford, 2016). One study measured arousal to image-and-text HWLs only on sugar-sweetened beverages (Mantzari et al., 2018) and four studies to text-only and image-and-text HWLs on alcohol and energy-dense food products (Clarke et al., 2020a, 2020b; Grummon et al., 2019; Wigg & Stafford, 2016). Pooled analysis of these data – comprising eight comparisons from five studies – showed a large increase in negative emotional arousal for HWLs compared to no label: SMD −1.28 (95% CI −1.50 to −1.06), n = 7710, I² = 93% (see Supplementary material 2, Figure 1). One study (Mantzari et al., 2018) reported data on negative emotional arousal as a mediator on selection and found negative emotional arousal mediated the impact of the image-and-text HWL on selection of sugar-sweetened beverages.

##### Intentions

Purchase or consumption intentions were measured in five studies focussed on non-alcoholic drinks (specifically sugar-sweetened beverages) (Bollard et al., 2016; Grummon et al., 2019; Roberto et al., 2016; VanEpps & Roberto, 2016) and alcoholic drinks (Wigg & Stafford, 2016). Pooled analysis of these data – comprising five comparisons from four studies and reverse coding those that concerned intentions *not to consume* – showed HWLs led to a reduction in intentions to purchase or consume: SMD −0.24 (95% CI −0.39 to −0.10), n = 4272, I² = 58% (see Supplementary material 2, Figure 2). One study was excluded from the pooled analysis as it provided insufficient detail (Bollard et al., 2016), not reporting results by group. This study reported that image-and-text and text-only HWLs significantly decreased the likelihood of buying sugar-sweetened beverages.

##### Acceptability

Five studies measured acceptability of HWLs. These studies were not suitable for meta-analysis due to the wide range of acceptability measures used and the lack of a control group. One study (Mantzari et al., 2018) found lower support for the introduction of image-and-text HWLs on sugar-sweetened beverages in the control group in which participants had not viewed a HWL, than in in the intervention group. Two studies (Roberto et al., 2016; VanEpps & Roberto, 2016) found that the majority of participants (73.3% (Roberto et al., 2016) and 62.7% (VanEpps & Roberto, 2016)) were in favour of text-only sugar-sweetened beverages warning label policies after viewing a text-only HWL, and this did not differ across experimental groups. Two studies (Clarke et al., 2020a, 2020b) compared image-and-text HWLs to text-only HWLs on food and alcohol products respectively. Both found acceptability was lower in the image-and-text HWL groups. One study (Stafford & Salmon, 2017) investigated acceptability of an alcoholic drink with a HWL in different label conditions and found significantly lower ratings for the image-and-text HWL compared to the control condition. There was no difference in this study between the control condition and the text-only HWL or between the text-only and image-and-text HWL.

## Discussion

### Summary of main results

This systematic review with meta-analysis provides initial evidence that HWLs placed on the packaging of food (including non-alcoholic drinks) and alcohol products have significant potential to reduce the selection of such products. Image-and-text and text-only HWLs were both effective in reducing selection compared to no label. This evidence is, however, limited in both quantity and quality. It is based nearly exclusively on studies conducted in artificial, primarily online, settings, using images of products requiring hypothetical selection, and assessing immediate impact after a single exposure to a label. None of the included studies were conducted in field settings – although one study was conducted in a naturalistic laboratory purposefully designed to resemble a real shopping environment – and none measured impact on actual consumption. Finally, in terms of the types of products, for the primary outcomes only two studies assessed the impact of HWLs on alcohol, and only two on food, with most of the evidence concerning non-alcoholic drinks (n = 9).

### Primary outcomes

Existing evidence suggests that placing image-and-text and text-only HWLs on food and alcohol products has a large effect in reducing selection of that product. Translating this effect size into a more familiar metric suggests a potential effect equivalent to a 26% decrease in the likelihood of selecting a product displaying a HWL, compared to products with no HWL. GRADE assessment indicated this evidence was of low certainty, meaning that further research is likely to change the effect estimate. This low rating reflects significant concerns for risk of bias in the studies included in the meta-analysis, a limited number of studies using HWLs on real products and an absence of evidence in field settings. Subgroup analyses suggested that image-and-text HWLs may have a larger effect than text-only HWLs, with the likelihood of selecting a product with an image-and-text HWL 14 percentage points lower (35% vs 21%) than selecting a product displaying a text-only HWL, although this difference was not statistically significant and so considerable uncertainty remains. There were no differences identified by product type, although there were insufficient data on alcohol and food products (not including non-alcoholic drinks) to have confidence in this.

Given the considerable heterogeneity in studies revealed in the meta-analysis, an additional exploratory subgroup analysis was conducted to investigate differential effects by setting. Categorisation by laboratory or online setting indicated substantial heterogeneity in the online studies, and lower heterogeneity in the laboratory studies. This is likely due to differences in products, label content and populations, as well as many studies with large samples producing narrow confidence intervals and a reduced likelihood of their overlap. There was also clear evidence of a large effect in online studies and some evidence of a small effect in laboratory studies, which was, however, not statistically significant, with wide confidence intervals. These confidence intervals suggest the possibility of a substantial effect favouring the intervention (24%) and a small effect favouring the control (7%). It is plausible that online studies misrepresent the magnitude of likely effects more than laboratory studies as they are carried out in distant, highly artificial settings, inevitably do not use physical products and may elicit focused attention on the label content that is less likely to occur in other contexts. However, evidence from laboratory settings is preliminary and limited to a small number of studies with small sample sizes relative to online settings. The only study using a naturalistic laboratory setting (Grummon et al., 2019) reported effects of HWLs of a comparable magnitude to online studies. This uncertainty demonstrates the need for replication of online experimental findings in more ecologically valid settings. Notably, no studies included in the review assessed the impact of HWLs in field settings using experimental designs. One field study that used a non-randomised design – thus ineligible for the current review – found image-and-text HWLs reduced purchases of sugar-sweetened beverages from 21.4% at baseline to 18.2%, with no effect of text-only HWLs (Donnelly et al., 2018).

No studies included in the current review assessed amount consumed although one study included speed of consumption, a measure that might predict total consumption (Stafford & Salmon, 2017).

### Secondary outcomes

Placing image-and-text or text-only HWLs on food and alcohol products elicits negative emotional arousal (Clarke et al., 2020a, 2020b; Grummon et al., 2019; Mantzari et al., 2018; Wigg & Stafford, 2016). Evidence from tobacco control indicates that stronger negative emotions increase the likelihood of quit attempts (Brewer et al., 2019; Cho et al., 2018), a finding supported by one of the studies in this review, which found negative emotional arousal mediated the effect of HWLs on selection of sugary drinks (Mantzari et al., 2018). Future studies could usefully examine the role of negative emotions to better understand these and other potential mechanisms by which HWLs exert their effects.

In five studies HWLs were found to reduce intentions to purchase or consume, or increase intentions to limit consumption of non-alcoholic drinks (Bollard et al., 2016; Grummon et al., 2019; Roberto et al., 2016; VanEpps & Roberto, 2016; Wigg & Stafford, 2016), which is in line with the large effects found on hypothetical selection. There is, however, a well-documented intention-behaviour gap, with a medium-to-large sized change in intentions leading only to a small-to-medium-sized change in behaviour (d = 0.36) (Webb & Sheeran, 2006), reiterating the need for replication of findings using valid behavioural outcomes.

A small number of studies suggest that text-only HWLs may be more acceptable than image-and-text HWLs (Clarke et al., 2020a, 2020b), despite the likely larger effect sizes of the latter. Communicating evidence of the effectiveness of HWLs could increase their acceptability, as might exposure to them (Donnelly et al., 2018). Importantly – and as with all secondary outcomes – there are inevitably other studies not included in the current review which assess acceptability of HWLs, a component key to the likelihood of an interventions’ implementation (Cullerton et al., 2016). Initial research on the acceptability of food and alcohol HWLs suggests those that show the greatest potential for reducing selection and consumption might also be the least acceptable (Pechey et al., 2020). Future focused research is needed to assess the acceptability of different HWLs on different products, and the extent to which acceptability is increased when these are presented alongside evidence of their effectiveness in changing behaviour to improve population health (Pechey et al., 2014; Reynolds et al., 2018).

### Quality of evidence

At the level of individual studies included in this review, most gave insufficient information to allow evaluation of their risk of bias. This is reflected in the majority of the summary risk of bias assessments being judged as having some concerns. For example, descriptions of attempts to address selection bias through randomisation and allocation procedures were often inadequate, although it was assumed by study authors that for the online studies these would have been carried out adequately through the online survey platform. Another key risk of bias domain concerned the selective reporting of results. Only three studies pre-registered protocols which outlined analysis intentions (Clarke et al., 2020a, 2020b; Grummon et al., 2019). Four of the included studies would have been rated as low risk of bias overall had they met the requirements of this domain (Ang et al., 2019; Mantzari et al., 2018; Roberto et al., 2016; VanEpps & Roberto, 2016). Future experimental studies in this field would benefit from following reported standard guidelines to reduce risk of bias, such as CONSORT, and by pre-registering protocols and analysis intentions. Pre-registering protocols – outlining research questions, study methods and analysis intentions – is an important step in increasing research transparency and reducing the potential for bias (Munafò, 2019). As outlined above, a global assessment of the evidence for the selection outcome, through applying the GRADE system, led to a rating of the evidence as low certainty, meaning that confidence in this effect estimate is limited, and that the true effect may be substantially different.

### Strengths and limitations

This review is novel, being the first to our knowledge to assess the impact on selection and consumption of HWLs placed on food and alcohol products. It is robust in its methods, being pre-registered, using Cochrane methods (Higgins et al., 2019) and is reported in line with PRISMA guidelines (Moher et al., 2009). A comprehensive and sensitive search strategy was developed, with multiple databases (including the grey literature) being searched, together with snowball searching. However, as with all systematic reviews, it remains possible that some eligible articles were missed. Additionally, only warnings that described health consequences were included in the review and not warnings relating to the specific content of foods such as high levels of a given nutrient. Future reviews could therefore consider assessing the potential effectiveness of a wider range of types of warning labels, and comparing these to HWLs. Relatively few studies – nine – are included in our primary meta-analysis, although meta-analyses often include fewer studies than this; three-quarters of meta-analyses within 22,000 Cochrane reviews contain five or fewer studies, with the median being three (Davey et al., 2011). Despite there being relatively few studies, a meta-analysis was considered the most appropriate way of summarising the data as planned meta-analyses are less subject to bias and are more transparent than other means of summarising quantitative data (Valentine et al., 2010). In addition, the sample size was very large (n = 12,635) and included generally highly powered component studies thus providing precise estimates.

### Implications of findings

Most existing evidence for the impact of HWLs is for their use on tobacco products (Hammond, 2011; Hammond et al., 2004). This review examines the evidence for their use in relation to food (including non-alcoholic drinks) and alcohol products, finding that most studies to date have targeted non-alcoholic drinks with only two studies on food and two on alcohol. Although the included studies encompassed a large number and wide range of participants, the relatively low number of studies included in the meta-analysis – while not a concern in and of itself – does reflect some of the key gaps in the evidence base. In particular, the findings are not readily generalisable beyond online and lab settings. There is a need for further studies using experimental designs and robust procedures at low risk of bias that assess the impact of HWLs on physical products in laboratory and field settings. Evidence is also needed for the impact of HWLs in use over sustained time-periods, and for the effects on actual consumption as well as selection. Future research should investigate the optimal content, whereas many of the studies included in this review simply investigate a HWL compared to no label. Additionally, different presentations of HWLs – such as their position on a product – may impact their effectiveness. One study in the current review, conducted in a naturalistic lab setting (Grummon et al., 2019) found that large labels, that covered branding, were effective in reducing selection. Another non-randomised study – not included in the review – found large HWLs presented on shelves were effective in a field setting (Donnelly et al., 2018). In both studies the HWLs were very clearly visible, with placement different to the likely placement if the labels were to be implemented. Further field studies investigating a different placements of the HWLs are required before any policy recommendations are made.

The results of this review are in accordance with those from other reviews on information-based choice architecture interventions. These show that information-based cues can influence selection and consumption of food and alcohol products (Carter et al., 2018), and nutritional labelling in the form of energy (calorie) labelling on food products reduces energy purchased (Crockett et al., 2018; Shangguan et al., 2019). The findings are also in line with a recent narrative review which outlined the extremely limited evidence-base for HWLs on alcohol (Hassan & Shiu, 2018). Encouragingly, image-and-text HWLs on tobacco products have demonstrated positive effects on quitting behaviour in field settings over longer periods (Brewer et al., 2016), and other forms of food labelling in real-world settings – such as recently implemented labels in Chile warning of high fat, sugar, and salt content – show a positive impact on unhealthy food selection (Araya et al., 2018). Expectations of an equivalent impact of HWLs on food and alcohol products should remain muted, however, until they are similarly tested.

## Conclusions

This review suggests the significant potential for decreasing selection of food and alcohol products of adding health warning labels that communicate adverse health-related consequences of consumption to the packaging of these products. However, the evidence included in the review had low overall certainty, meaning that confidence in the estimated effect is limited. While the size of the effect was estimated to be a 26% (ranging from 20% to 32%) reduction in likelihood of selection, this was derived nearly exclusively from studies conducted in artificial laboratory or online settings, with outcomes assessed immediately after a single exposure. Studies in field and more naturalistic laboratory settings – assessing consumption as well as selection – are urgently needed to enable more generalisable and accurate estimation of real-world effects.

## Supplementary Material

Supplemental Material

## Data Availability

The registered data can be found on the Open Science Framework (https://osf.io/kmqut/) which is linked to the PROSPERO international Prospective Register of Systematic Reviews database (PROSPERO registration number: CRD42018106522). All meta-analysed data can be found in the Supplementary Material and the full meta-analysis outputs are available in the forest plots in Figures 2 –5 and the Supplementary Material (Figures 1 and 2).
