# Supplementary material for: Impact of health warning labels on selection and consumption of food and alcohol products: systematic review with meta-analysis
Source: Health Psychol Rev. 2020 Jul 2;15(3):430–53. doi: 10.1080/17437199.2020.1780147 (PMC8635708; doi:10.1080/17437199.2020.1780147)
Supplement: Supplemental Material [file RHPR_A_1780147_SM8962.docx]

**Supplementary material**

**S.1.** *Table 1. Author Judgement, and Support for Judgement, of Risk of Bias in Included Studies (for domains deemed important to current intervention)*

| Studies | Random sequence generation | Allocation concealment | Blinding of participants and personnel | Blinding of outcome assessment | Incomplete outcome data | Selective reporting | Baseline comparability | Summary risk of bias |
| --- | --- | --- | --- | --- | --- | --- | --- | --- |
| Ang 2019 | Some concerns  “Those who consented were randomized into 1 of the 3 arms”- insufficient information on how the randomisation method was conducted. | Low  Insufficient details to enable judgement, but assume allocation was conducted independently via online platform | Low  There was not an attempt to formally blind but we judged it unlikely participants would ascertain the nature of the other conditions | Low  Blinding not stated but assume outcome data was collected objectively via online platform | Low  Outcome data available for all participants | Some Concerns  Unable to find any pre-registered analysis plans | Low  Even group sizes but statistical differences between groups on baseline characteristics not reported | Some concerns |
| Acton 2018 | Some Concerns  “Participants were randomly allocated”'- insufficient information on how the randomisation method was conducted. | Low  Insufficient details to enable judgement, but assume allocation was conducted independently via online platform | Low  There was not an attempt to formally blind but we judged it unlikely participants would ascertain the nature of the other conditions | Low  Blinding not stated but assume outcome data was collected objectively via online platform | Low  Outcome data available for nearly all participants | Some Concerns  Unable to find any trial registration or published protocol | Low  No observed differences across conditions | Some Concerns |
| Billich 2018 | Low  “The randomisation was done using a client side JavaScript randomisation, selecting a random number between one and five” | Some concerns  Insufficient details to enable judgement, assume allocation was conducted independently via online platform but uneven groups sizes suggest bias from allocation procedure. | Low  There was not an attempt to formally blind but we judged it unlikely participants would ascertain the nature of the other conditions | Low  Blinding not stated but assume outcome data was collected objectively via online platform | Low  Outcome data missing for high proportion of participants, but proportions of missing data similar across groups | Some Concerns  Unable to find any trial registration or published protocol | Low  Uneven group sizes but no observed differences across conditions | Some Concerns |
| Clarke 2020a | Low  Participants randomised independently via online survey platform | Low  Allocation was conducted independently via online platform | Low  There was not an attempt to formally blind but we judged it unlikely participants would ascertain the nature of the other conditions | Low  Blinding not stated but outcome data was collected objectively via online platform | Low  836 participants dropped out (12%), unclear whether pre or post randomisation | Low  Protocol and pre-registered analysis plan available | Low  Even group sizes, no differences between groups | Low |
| Clarke 2020b | Low  Participants randomised independently via online survey platform | Low  Allocation was conducted independently via online platform | Low  There was not an attempt to formally blind but we judged it unlikely participants would ascertain the nature of the other conditions | Low  Blinding not stated but outcome data was collected objectively via online platform | Low  315 participants dropped out (7%), unclear whether pre or post randomisation | Low  Protocol and pre-registered analysis plan available | Low  Even group sizes, no differences between groups | Low |
| Grummon 2019 | Low  Participants randomised independently | Low  Allocation was conducted through a pre-specified independent allocation | Low  There was an attempt to blind participants through a cover story and authors highlight few participants guessed study aims | Low  Not reported but likely researchers would be aware of condition, but were not present for task and outcome was objective. | Low  Outcome data reported for all participants | Low  Protocol and pre-registered analysis plan available | Low  Even group sizes, no differences between groups | Low |
| Mantzari 2018 | Low  Participants randomised independently via online survey platform | Low  Insufficient details to enable judgement, but assume allocation was conducted independently via online platform. | Low  There was not an attempt to formally blind but we judged it unlikely participants would ascertain the nature of the other conditions | Low  Blinding not stated but assume outcome data was collected objectively via online platform | Low  377 (16%) participants dropped out, unclear whether this was pre or post randomisation | Some Concerns  Unable to find any trial registration or published protocol | Low  No observed differences across conditions | Some Concerns |
| Mantzari 2020 | Low  “Randomisation was stratified according to SEP to ensure a balance between groups and was performed by a statistician independent of the research team” | Low  Bias low as allocation carried out independently of the research team (by statistician). | Low  Participants given a cover story to disguise real aims. Highly unlikely researchers could have been blinded | Some Concerns  Not reported explicitly but likely researchers delivering interventions and measuring outcomes would have been aware of/setting up the intervention conditions | Low  Outcome data available for nearly all participants | Some Concerns  Unable to find any trial registration or published protocol | Low  No observed differences across conditions | Some Concerns |
| Roberto 2016 | Some Concerns  “Randomly assigned to one of 6 conditions” - insufficient information on how the randomisation method was conducted. | Low  Insufficient details to enable judgement, but assume allocation was conducted independently via online platform. | Low  There was not an attempt to formally blind but we judged it unlikely participants would ascertain the nature of the other conditions | Low  Blinding not stated but assume outcome data was collected objectively via online platform | Low  Outcome data available for nearly all participants | Some Concerns  Unable to find any trial registration or published protocol | Some Concerns  Baseline differences not reported | Some Concerns |
| Stafford 2017 | Some Concerns  “Participants were randomly allocated”- insufficient information on how the randomisation method was conducted. | Some Concerns  Not described | Low  Researchers attempted to conceal real aim of study, but didn't report on number of participants who guessed aim | Some Concerns  Not reported but researchers were recording consumption speed so it is highly likely they would be aware of condition | Low  No missing outcome data | Some Concerns  Unable to find any trial registration or published protocol | Low  No baseline differences between groups | Some Concerns |
| Temple 2015 | Some Concerns  Within subjects design, order of conditions was randomised (not reported in paper but author was contacted) and no further information given about method. | Some Concerns  Highly likely that researchers would not be blinded and would be aware of study groups | Some Concerns  Participants were given a cover story, but likely with a within subjects design with the 2nd and 3rd time participants will guess the differences- participants told study was on how different factors might influence purchasing | Some Concerns  No details given to enable judgement | Some Concerns  Number of participants in final analysis not explicitly stated, but assume it is the full sample | Some Concerns  Unable to find any trial registration or published protocol | Low  No evidence of differences between groups | Some Concerns |
| VanEpps 2016 | Some Concerns  “Randomly assigned to one of 6 conditions” - insufficient information on how the randomisation method was conducted. | Low  Insufficient details to enable judgement, but assume allocation was conducted independently via online platform. | Low  There was not an attempt to formally blind but we judged it unlikely participants would ascertain the nature of the other conditions | Low  Blinding not stated but assume outcome data was collected objectively via online platform | Low  Outcome data available for nearly all participants | Some Concerns  Unable to find any trial registration or published protocol | Low  No observed differences across conditions | Some Concerns |

**S.2.**

*
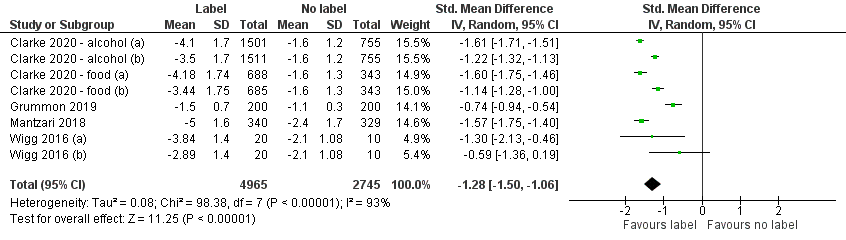
*

*Figure 1. Forest plot for secondary outcome of negative emotional arousal*


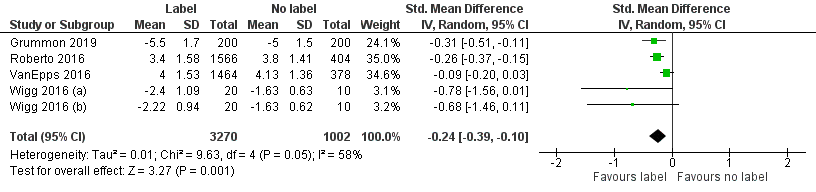


*Figure 2. Forest plot for secondary outcome of intentions*

**S.3. Searches**

**All searches combined = 8421**

**Removing duplicates = 6364**

**MEDLINE SEARCH - OVID MEDLINE (R) and In process& other non-indexed citations 1946 to present**

**Ran on 16.9.19**

**Records returned =** 3583

1. *Food/
2. *Food intake/
3. *Food habits/
4. *Food preferences/
5. *Eating/
6. (eat$ or ate or low-fat or meal$1 or dessert$1 or snack$ or fat$1 or sugar$1 or salt$1 or soft drink$1 or sugar sweetened beverage$1 or sugary drink$1 or SSB$1).ti, ab. /
7. *Beverages/
8. *Alcohol drinking/
9. *Drinking/
10. (drink$ or drunk or alcohol or beverage$1 or beer$1 or lager$1 or wine$1 or cider$1 or alcopop$1 or alco-pop$1 or spirit$1 or liquor$1 or liquer$1 or liqueur$1 or whisky or whiskey or whiskies or whiskeys or schnapp$1 or brandy or brandies or gin$1 or rum$1 or tequila$1 or vodka$1 or cocktail$1).ti, ab/
11. 1 or 2 or 3 or 4 or 5 or 6 or 7 or 8 or 9 or 10
12. *Product labelling/
13. ((picture$1 or pictorial or image$ or graphic$ or text$ or written or health$ or ill$ or disease$1 or adverse or aversive) adj8 (warning$1 or messag$ or label$ or statement$1 or pack$)).ti,ab. /
14. 12 or 13
15. 11 and 14
16. exp animals/ not humans/ 15 not 16
17. (editorial or case reports or letter or comment).mp. [mp=title, abstract, original title, name of substance word, subject heading word, floating sub-heading word, keyword heading word, protocol supplementary concept word, rare disease supplementary concept word, unique identifier, synonyms] / 17 not 18

**EMBASE (OvidSP) 1974 to 2019**

**Ran on 16.9.19**

**Recorded returned: 1960**

1. *Food/
2. *Food intake/
3. *Food habits/
4. *Food preferences/
5. *Eating/
6. (eat$ or ate or low-fat or meal$1 or dessert$1 or snack$ or fat$1 or sugar$1 or salt$1 or soft drink$1 or sugar sweetened beverage$1 or sugary drink$1 or SSB$1).ti, ab. /
7. *Beverages/
8. *Alcohol drinking/
9. *Drinking/
10. (drink$ or drunk or alcohol or beverage$1 or beer$1 or lager$1 or wine$1 or cider$1 or alcopop$1 or alco-pop$1 or spirit$1 or liquor$1 or liquer$1 or liqueur$1 or whisky or whiskey or whiskies or whiskeys or schnapp$1 or brandy or brandies or gin$1 or rum$1 or tequila$1 or vodka$1 or cocktail$1).ti, ab/
11. 1 or 2 or 3 or 4 or 5 or 6 or 7 or 8 or 9 or 10
12. *Labelling/
13. ((picture$1 or pictorial or image$ or graphic$ or text$ or written or health$ or ill$ or disease$1 or adverse or aversive) adj8 (warning$1 or messag$ or label$ or statement$1 or pack$)).ti,ab. /
14. 12 or 13
15. 11 and 14
16. exp animals/ not humans/
17. 15 not 16
18. (editorial or case reports or letter or comment).mp. [mp=title, abstract, original title, name of substance word, subject heading word, floating sub-heading word, keyword heading word, protocol supplementary concept word, rare disease supplementary concept word, unique identifier, synonyms] /
19. 17 not 18

**PsycINFO (EBSCOhost) 1806 to 2019**

**Ran on 16.9.19**

**Records returned = 1118**

S15 S11 AND S14

S14 S12 OR S13

S13 (picture$1 or pictorial or image$ or graphic$ or text$ or written or health$ or ill$ or disease$1 or adverse or aversive ) AND ( warning$1 or messag$ or label$ or statement$1 or pack$)

S12 *Product labelling/

S11 (drink$ or drunk or alcohol or beverage$1 or beer$1 or lager$1 or wine$1 or cider$1 or alcopop$1 or alco-pop$1 or spirit$1 or liquor$1 or liquer$1 or liqueur$1 or whisky or whiskey or whiskies or whiskeys or schnapp$1 or brandy or brandies or gin$1 or rum$1 or tequila$1 or vodka$1 or cocktail$1.ti, ab) AND (S1 OR S2 OR S3 OR S4 OR S5 OR S6 OR S7 OR S8 OR S9 OR S10)

S10 drink$ or drunk or alcohol or beverage$1 or beer$1 or lager$1 or wine$1 or cider$1 or alcopop$1 or alco-pop$1 or spirit$1 or liquor$1 or liquer$1 or liqueur$1 or whisky or whiskey or whiskies or whiskeys or schnapp$1 or brandy or brandies or gin$1 or rum$1 or tequila$1 or vodka$1 or cocktail$1.ti, ab

S9 *Drinking/

S8 *Alcohol drinking/

S7 *Beverages/

S6 eat$ or ate or low-fat or meal$1 or dessert$1 or snack$ or fat$1 or sugar$1 or salt$1 or soft drink$1 or sugar sweetened beverage$1 or sugary drink$1 or SSB$1.ti,ab

S5 *Eating/

S4 *Food preferences/

S3 *Food habits/

S2 *Food intake/

S1 *Food/

**Cochrane Central Register of Controlled Trials (CENTRAL), 1992 to 3^rd^ March 2016 (Issue 2 of 12, 2016)**

**Ran on 16.9.19**

**Records returned = 123**

#1 eat* or ate or low-fat or meal* or dessert* or snack* or fat* or sugar* or salt* or soft drink* or sugar sweetened beverage* or sugary drink* or SSB* or drink* or drunk or alcohol or beverage* or beer* or lager* or wine* or cider* or alcopop* or alco-pop* or spirit* or liquor* or liquer* or liqueur* or whisky or whiskey or whiskies or whiskeys or schnapp* or brandy or brandies or gin* or rum* or tequila* or vodka* or cocktail*

#2 (picture* or pictorial or image* or graphic* or text* or written or health* or ill* or disease* or adverse or aversive) near8 (warning* or messag* or label* or statement* or pack*)

#3 1 and 2

#4 rat or rats or mouse or mice or murine or rodent or rodents or hamster or hamsters or pig or pigs or porcine or rabbit or rabbits or animal or animals or dog or dogs or cat or cats or cow or cows or bovine or sheep or ovine or monkey or monkeys

#5 2 not 3

**Web of Science**

- **Science Citation Index Expanded, 1900 to 2019;**
- **Social Sciences Citation Index, 1956 to 2019;**
- **Conference Proceedings Citation Index - Science, 2019;**
- **Conference Proceedings Citation Index - Social Science & Humanities, 1990 to 2019**

**Ran on 16.9.19**

**Records returned =** 1, 637

# 1

TS = ~(eat* or ate or low-fat or meal* or dessert* or snack* or fat* or sugar* or salt* or soft drink* or sugar sweetened beverage* or sugary drink* or SSB* or drink* or drunk or alcohol or beverage* or beer* or lager* or wine* or cider* or alcopop* or alco-pop* or spirit* or liquor* or liquer* or liqueur* or whisky or whiskey or whiskies or whiskeys or schnapp* or brandy or brandies or gin* or rum* or tequila* or vodka* or cocktail*)

Indexes=SCI-EXPANDED, SSCI, CPCI-S, CPCI-SSH Timespan=All years

# 2

TS= ((picture* or pictorial or image* or graphic* or text* or written or health* or ill* or disease* or adverse or aversive) NEAR/8 (warning* or messag* or label* or statement* or pack*))

Indexes=SCI-EXPANDED, SSCI, CPCI-S, CPCI-SSH Timespan=All years

# 3

#2 and #1

Indexes=SCI-EXPANDED, SSCI, CPCI-S, CPCI-SSH Timespan=All years

# 4

TS=(rat or rats or mouse or mice or murine or rodent or rodents or hamster or hamsters or pig or pigs or porcine or rabbit or rabbits or animal or animals or dog or dogs or cat or cats or cow or cows or bovine or sheep or ovine or monkey or monkeys)

Indexes=SCI-EXPANDED, SSCI, CPCI-S, CPCI-SSH Timespan=All years

# 5

#3 NOT #4

Indexes=SCI-EXPANDED, SSCI, CPCI-S, CPCI-SSH Timespan=All years

# 6

3 NOT #4

Refined by: [excluding] WEB OF SCIENCE CATEGORIES: ( FOOD SCIENCE TECHNOLOGY OR PSYCHIATRY OR ENDOCRINOLOGY METABOLISM OR BIOCHEMISTRY MOLECULAR BIOLOGY OR ENVIRONMENTAL SCIENCES OR GASTROENTEROLOGY HEPATOLOGY OR PHARMACOLOGY PHARMACY OR ONCOLOGY OR CLINICAL NEUROLOGY OR CARDIAC CARDIOVASCULAR SYSTEMS OR PEDIATRICS OR DENTISTRY ORAL SURGERY MEDICINE OR NEUROSCIENCES OR SURGERY OR RADIOLOGY NUCLEAR MEDICINE MEDICAL IMAGING OR MATERIALS SCIENCE MULTIDISCIPLINARY OR IMMUNOLOGY OR PERIPHERAL VASCULAR DISEASE OR BIOTECHNOLOGY APPLIED MICROBIOLOGY OR MICROBIOLOGY OR PLANT SCIENCES OR INFECTIOUS DISEASES OR CHEMISTRY APPLIED OR CHEMISTRY PHYSICAL OR CELL BIOLOGY OR SPORT SCIENCES OR CHEMISTRY ANALYTICAL OR ENGINEERING ELECTRICAL ELECTRONIC OR HEALTH CARE SCIENCES SERVICES OR NURSING OR TOXICOLOGY OR WATER RESOURCES OR GENETICS HEREDITY OR ENGINEERING CHEMICAL OR ENGINEERING ENVIRONMENTAL OR OBSTETRICS GYNECOLOGY OR UROLOGY NEPHROLOGY OR HEMATOLOGY OR RESPIRATORY SYSTEM OR HEALTH POLICY SERVICES OR BIOCHEMICAL RESEARCH METHODS OR GERIATRICS GERONTOLOGY OR AGRONOMY OR RHEUMATOLOGY OR CHEMISTRY ORGANIC OR BIOPHYSICS OR REHABILITATION OR AGRICULTURE MULTIDISCIPLINARY OR PHYSICS APPLIED OR PSYCHOLOGY DEVELOPMENTAL OR ENGINEERING MECHANICAL OR PATHOLOGY OR SOCIAL SCIENCES BIOMEDICAL OR ENGINEERING BIOMEDICAL OR DERMATOLOGY OR BIOLOGY OR POLYMER SCIENCE OR HORTICULTURE OR CHEMISTRY MEDICINAL OR GEOSCIENCES MULTIDISCIPLINARY OR SOCIAL SCIENCES INTERDISCIPLINARY OR VETERINARY SCIENCES OR NANOSCIENCE NANOTECHNOLOGY OR EDUCATION EDUCATIONAL RESEARCH OR OPTICS OR ECOLOGY OR ORTHOPEDICS OR AGRICULTURE DAIRY ANIMAL SCIENCE OR GERONTOLOGY OR ENGINEERING CIVIL OR CRITICAL CARE MEDICINE OR MARINE FRESHWATER BIOLOGY OR ENERGY FUELS OR COMPUTER SCIENCE ARTIFICIAL INTELLIGENCE OR MEDICAL LABORATORY TECHNOLOGY OR TRANSPLANTATION OR COMPUTER SCIENCE INTERDISCIPLINARY APPLICATIONS OR TROPICAL MEDICINE OR FAMILY STUDIES OR VIROLOGY OR COMPUTER SCIENCE THEORY METHODS OR CHEMISTRY INORGANIC NUCLEAR OR METALLURGY METALLURGICAL ENGINEERING OR SOIL SCIENCE OR SOCIOLOGY OR FISHERIES OR INTEGRATIVE COMPLEMENTARY MEDICINE OR PHYSICS CONDENSED MATTER OR COMPUTER SCIENCE INFORMATION SYSTEMS OR EDUCATION SCIENTIFIC DISCIPLINES OR INSTRUMENTS INSTRUMENTATION OR ANTHROPOLOGY OR OPHTHALMOLOGY OR OTORHINOLARYNGOLOGY OR SOCIAL WORK OR ENTOMOLOGY OR MECHANICS OR MEDICINE LEGAL OR SPECTROSCOPY OR PARASITOLOGY OR ALLERGY OR PRIMARY HEALTH CARE OR ELECTROCHEMISTRY OR ZOOLOGY OR ENGINEERING MULTIDISCIPLINARY OR CRYSTALLOGRAPHY OR IMAGING SCIENCE PHOTOGRAPHIC TECHNOLOGY OR PHYSICS ATOMIC MOLECULAR CHEMICAL OR EMERGENCY MEDICINE OR WOMEN S STUDIES OR RELIGION OR ANESTHESIOLOGY OR GEOCHEMISTRY GEOPHYSICS OR BUSINESS OR METEOROLOGY ATMOSPHERIC SCIENCES OR ENGINEERING MANUFACTURING OR MEDICAL INFORMATICS OR AGRICULTURAL ENGINEERING OR MATERIALS SCIENCE CHARACTERIZATION TESTING OR TRANSPORTATION OR MATERIALS SCIENCE BIOMATERIALS OR ENGINEERING INDUSTRIAL OR REMOTE SENSING OR TELECOMMUNICATIONS OR MATHEMATICAL COMPUTATIONAL BIOLOGY OR MANAGEMENT OR GREEN SUSTAINABLE SCIENCE TECHNOLOGY OR REPRODUCTIVE BIOLOGY OR OCEANOGRAPHY OR NUCLEAR SCIENCE TECHNOLOGY OR COMMUNICATION OR PHYSICS MULTIDISCIPLINARY OR DEVELOPMENTAL BIOLOGY OR AUTOMATION CONTROL SYSTEMS OR SOCIAL ISSUES OR COMPUTER SCIENCE SOFTWARE ENGINEERING OR STATISTICS PROBABILITY OR PSYCHOLOGY BIOLOGICAL OR CONSTRUCTION BUILDING TECHNOLOGY OR MATHEMATICS APPLIED OR NEUROIMAGING OR ACOUSTICS OR INFORMATION SCIENCE LIBRARY SCIENCE OR CRIMINOLOGY PENOLOGY OR MATERIALS SCIENCE COATINGS FILMS OR OPERATIONS RESEARCH MANAGEMENT SCIENCE OR THERMODYNAMICS OR MATHEMATICS INTERDISCIPLINARY APPLICATIONS OR FORESTRY OR MATERIALS SCIENCE COMPOSITES OR MATERIALS SCIENCE TEXTILES OR HOSPITALITY LEISURE SPORT TOURISM OR LAW OR ASTRONOMY ASTROPHYSICS OR COMPUTER SCIENCE HARDWARE ARCHITECTURE OR BIODIVERSITY CONSERVATION OR HISTORY PHILOSOPHY OF SCIENCE OR GEOGRAPHY PHYSICAL OR EVOLUTIONARY BIOLOGY OR MATERIALS SCIENCE CERAMICS OR HISTORY OR TRANSPORTATION SCIENCE TECHNOLOGY OR ETHICS OR ENGINEERING AEROSPACE OR GEOGRAPHY OR LIMNOLOGY OR PLANNING DEVELOPMENT OR MATERIALS SCIENCE PAPER WOOD OR AGRICULTURAL ECONOMICS POLICY OR PHYSICS MATHEMATICAL OR MYCOLOGY OR POLITICAL SCIENCE OR ANATOMY MORPHOLOGY OR LINGUISTICS OR GEOLOGY OR AREA STUDIES OR DEMOGRAPHY OR COMPUTER SCIENCE CYBERNETICS OR PSYCHOLOGY EDUCATIONAL OR SOCIAL SCIENCES MATHEMATICAL METHODS OR PSYCHOLOGY PSYCHOANALYSIS OR CELL TISSUE ENGINEERING OR MINERALOGY OR EDUCATION SPECIAL OR MINING MINERAL PROCESSING OR MATHEMATICS OR PHYSICS FLUIDS PLASMAS OR MICROSCOPY OR ENGINEERING GEOLOGICAL OR ROBOTICS OR HUMANITIES MULTIDISCIPLINARY OR MEDICAL ETHICS OR LANGUAGE LINGUISTICS OR PHYSICS PARTICLES FIELDS OR AUDIOLOGY SPEECH LANGUAGE PATHOLOGY OR BUSINESS FINANCE OR ETHNIC STUDIES OR ENGINEERING OCEAN OR PALEONTOLOGY OR URBAN STUDIES OR ENGINEERING MARINE OR ENGINEERING PETROLEUM OR INTERNATIONAL RELATIONS OR HISTORY OF SOCIAL SCIENCES OR ORNITHOLOGY OR CULTURAL STUDIES OR ANDROLOGY OR PUBLIC ADMINISTRATION OR PHYSICS NUCLEAR OR PHILOSOPHY OR ARCHAEOLOGY OR INDUSTRIAL RELATIONS LABOR OR LITERARY THEORY CRITICISM OR ART OR LITERATURE OR MUSIC OR PSYCHOLOGY MATHEMATICAL OR ASIAN STUDIES OR ARCHITECTURE OR FILM RADIO TELEVISION OR LOGIC OR MEDIEVAL RENAISSANCE STUDIES OR LITERATURE ROMANCE OR LITERATURE GERMAN DUTCH SCANDINAVIAN OR CLASSICS OR LITERATURE BRITISH ISLES OR LITERATURE SLAVIC OR THEATER OR FOLKLORE OR LITERATURE AMERICAN OR LITERATURE AFRICAN AUSTRALIAN CANADIAN OR POETRY OR DANCE OR LITERARY REVIEWS )

Indexes=SCI-EXPANDED, SSCI, CPCI-S, CPCI-SSH Timespan=All years

These exclusions mean that the search is focused on the following:

Searching in

MEDICINE GENERAL INTERNAL

MEDICINE RESEARCH EXPERIMENTAL

PSYCHOLOGY SOCIAL

NUTRITION DIETETICS

ECONOMICS

PUBLIC ENVIRONMENTAL OCCUPATIONAL HEALTH

PSYCHOLOGY MULTIDISCIPLINARY

PSYCHOLOGY EXPERIMENTAL

MULTIDISCIPLINARY SCIENCES

PSYCHOLOGY

PSYCHOLOGY APPLIED

SUBSTANCE ABUSE

BEHAVIORAL SCIENCES

ENVIRONMENTAL STUDIES

PSYCHOLOGY CLINICAL

PHYSIOLOGY

ERGONOMICS

**PsyArXiv**

Ran on 7^th^ October 2019

Records returned: 25

(food* or eat* or ate or low-fat or meal* or dessert* or snack* or fat* or sugar* or salt* or soft drink* or sugar sweetened beverage* or sugary drink* or SSB* or drink* or drunk or alcohol* or beverage* or beer* or lager* or wine* or cider* or alcopop* or alco-pop* or spirit* or liquor* or liquer* or liqueur* or whisky or whiskey or whiskies or whiskeys or schnapp* or brandy or brandies or gin* or rum* or tequila* or vodka* or cocktail*) AND (picture* or pictorial or image* or graphic* or text* or written or health* or ill* or disease* or adverse or aversive) near8 (warning* or messag* or label* or statement* or pack*)

**S4.**

**Labels used in included studies**

**
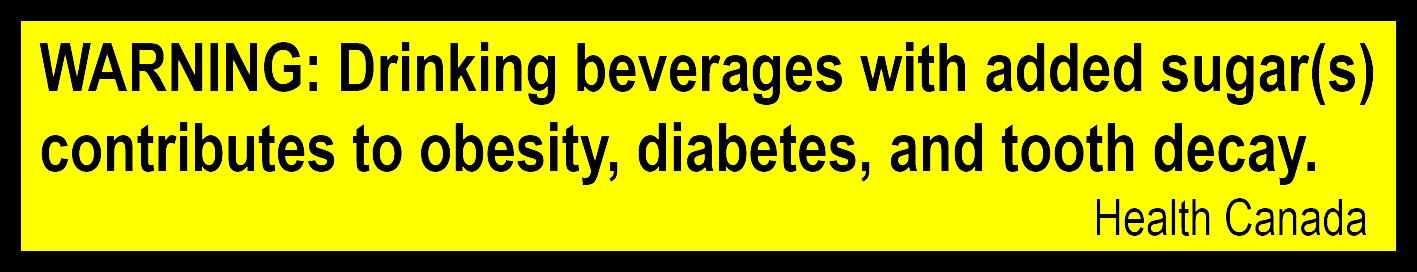
Acton (2017)**

**Ang (2019)**


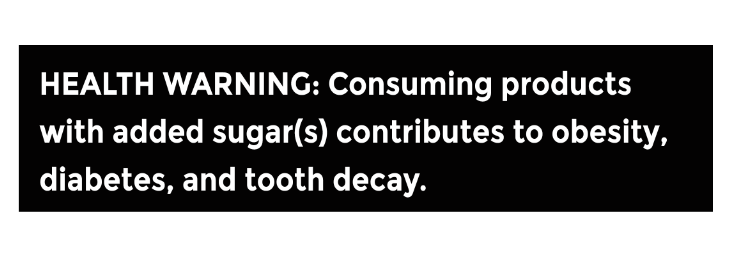


**Billich (2018)**

**
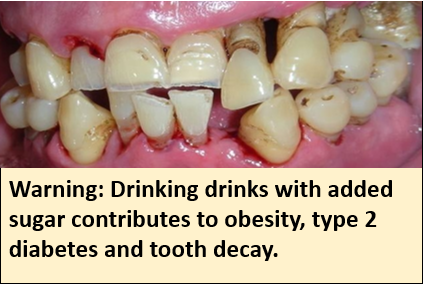
**

**
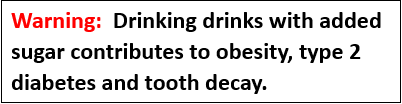
**

**Bollard (2016)**


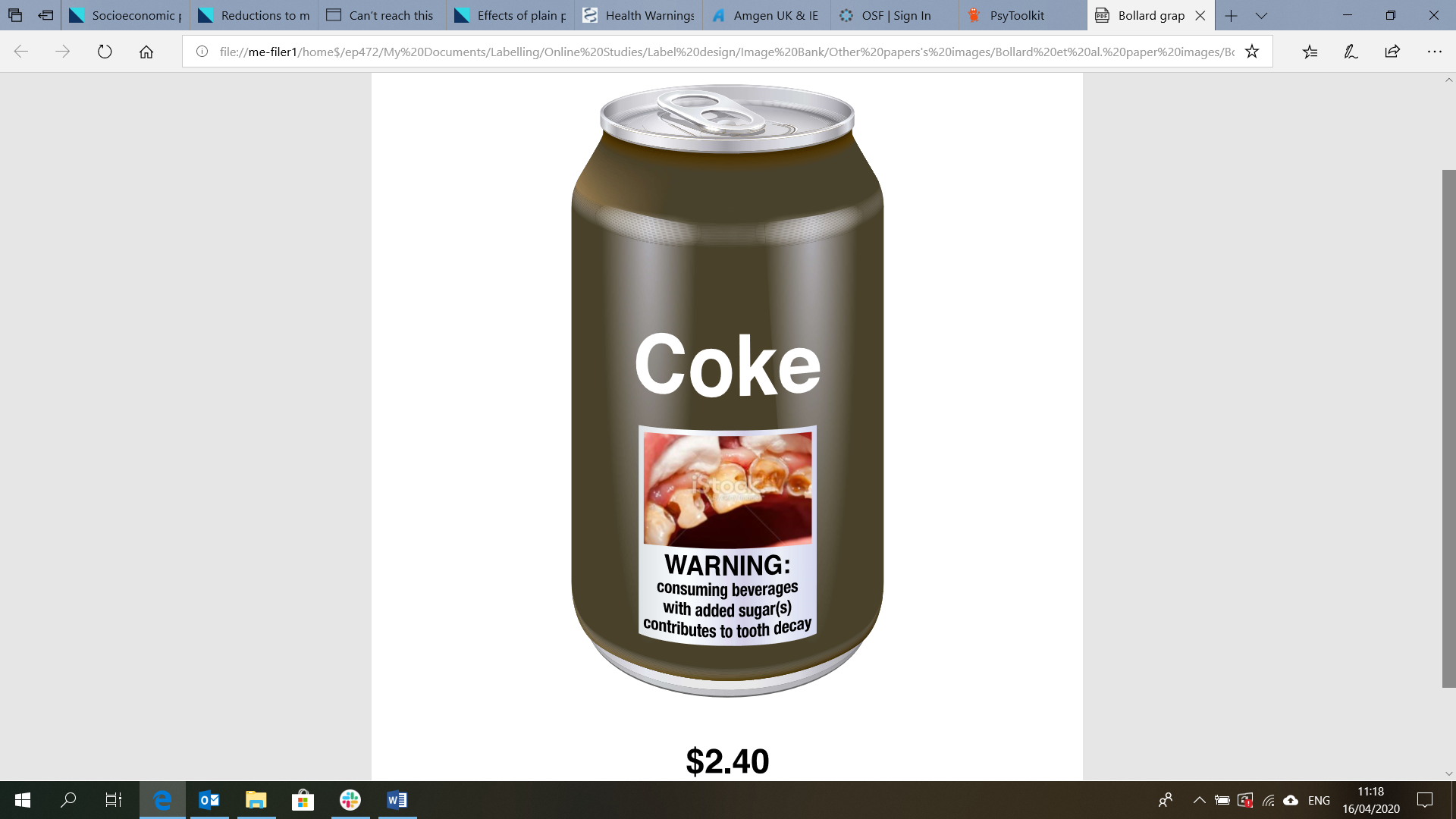


**Clarke – alcohol (2020)**

**
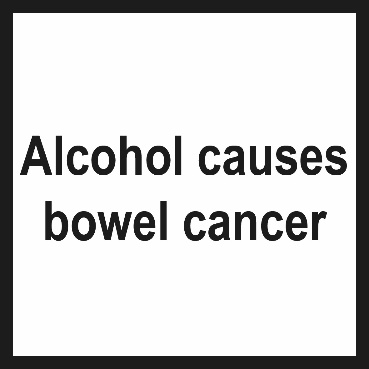

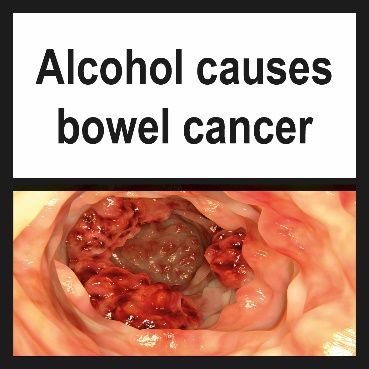
**

**Clarke – food (2020)**

**
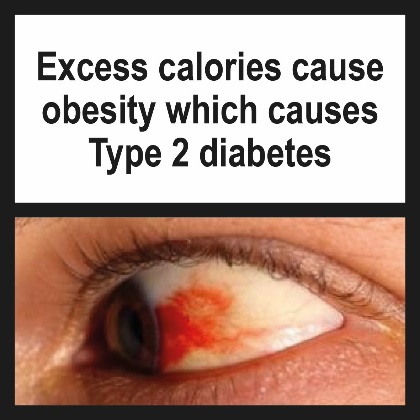

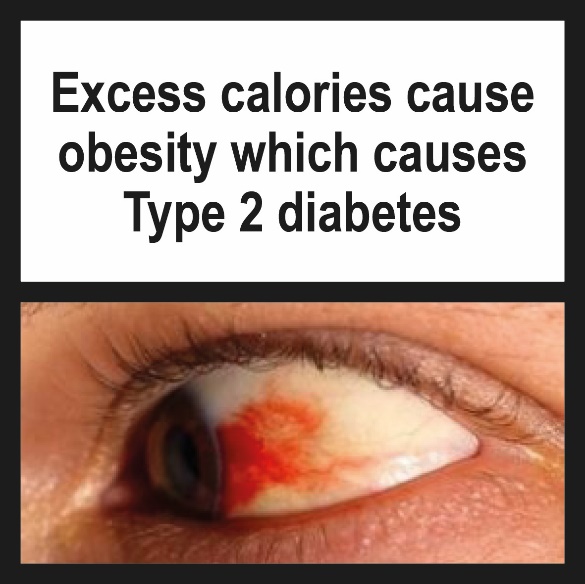
**

**Grummon (2019)**

**
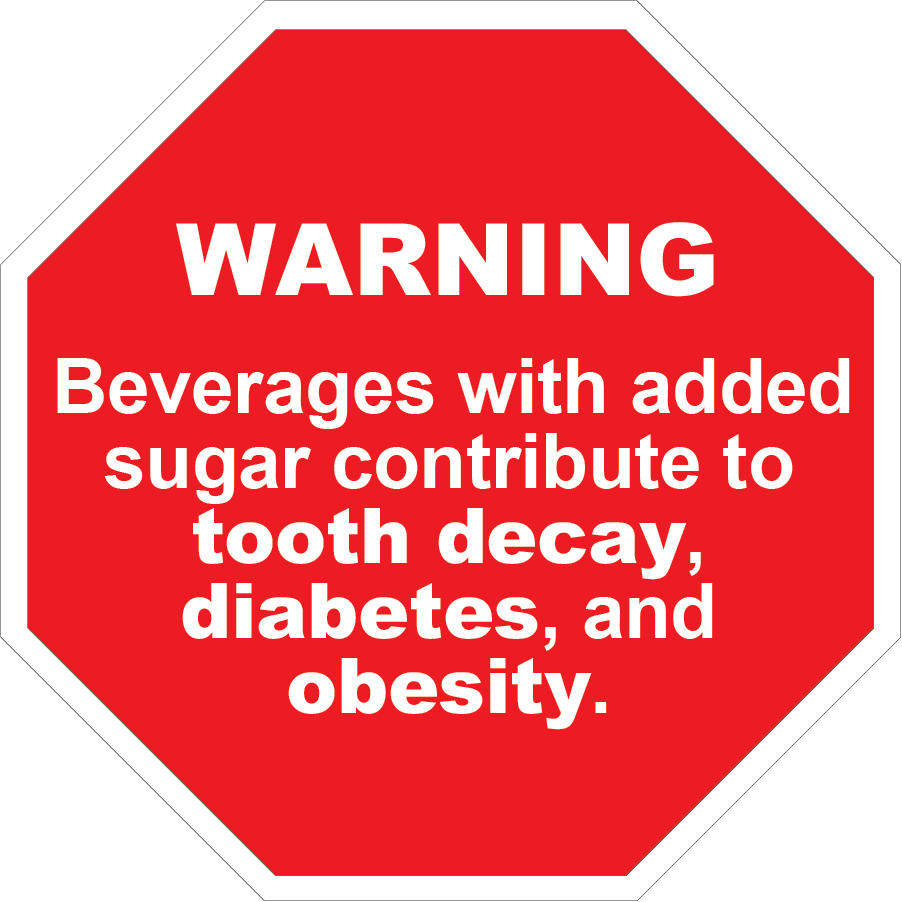
**

**Mantzari (2018) and Mantzari (2020)**


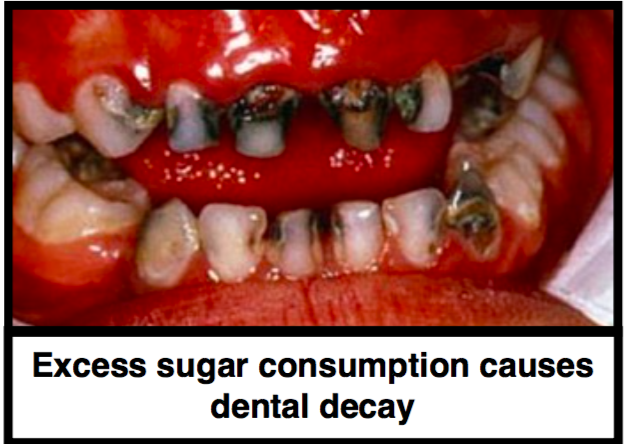


**Roberto (2016)**


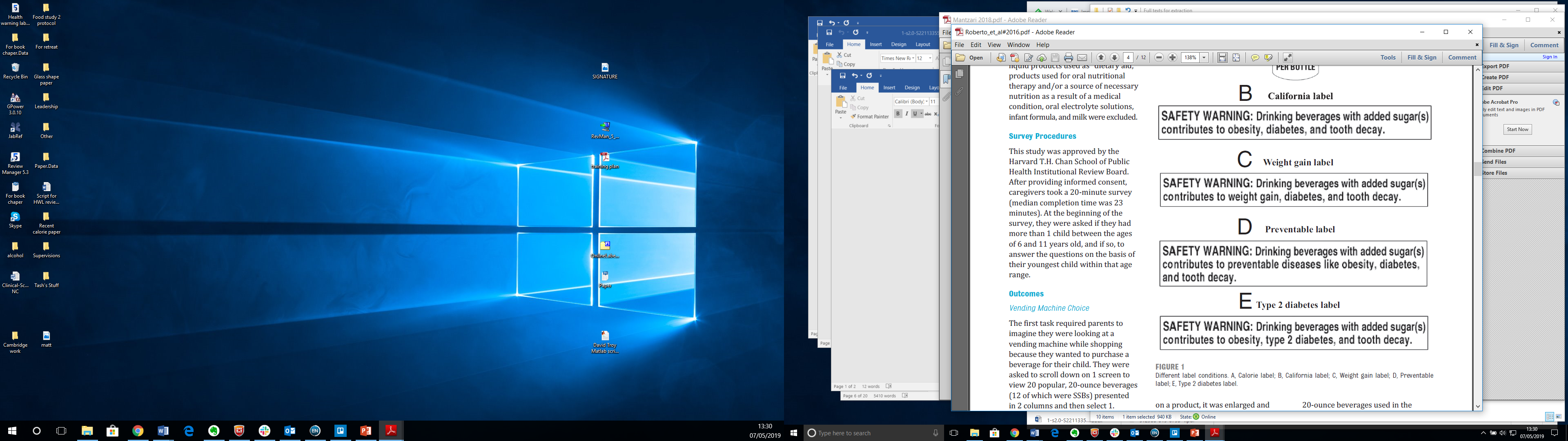


**Wigg & Stafford (2016) and Stafford & Salmon (2017)**


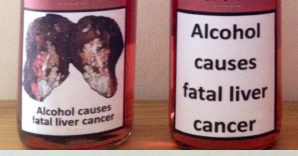


**VanEpps (2016)**
